# Multiyear Rehospitalization Rates and Hospital Outcomes in an Integrated Health Care System

**DOI:** 10.1001/jamanetworkopen.2019.16769

**Published:** 2019-12-04

**Authors:** Gabriel J. Escobar, Colleen Plimier, John D. Greene, Vincent Liu, Patricia Kipnis

**Affiliations:** 1 Systems Research Initiative, Kaiser Permanente Division of Research, Oakland, California; 2 Intensive Care Unit, Kaiser Permanente Medical Center, Santa Clara, California; 3 TPMG Consulting Services, Oakland, California

## Abstract

**Importance:**

Since the introduction of the rehospitalization rate as a quality measure, multiple changes have taken place in the US health care delivery system. Interpreting rehospitalization rates without taking a global view of these changes and new data elements from comprehensive electronic medical records yields a limited assessment of the quality of care.

**Objective:**

To examine hospitalization outcomes from a broad perspective, including the implications of numerator and denominator definitions, all adult patients with all diagnoses, and detailed clinical data.

**Design, Setting, and Participants:**

This cohort study obtained data from 21 hospitals in Kaiser Permanente Northern California (KPNC), an integrated health care delivery system that serves patients with Medicare Advantage plans, Medicaid, and/or Kaiser Foundation Health Plan. The KPNC electronic medical record system was used to capture hospitalization data for adult patients who were 18 years of age or older; discharged from June 1, 2010, through December 31, 2017; and hospitalized for reasons other than childbirth. Hospital stays for transferred patients were linked using public and internal sources.

**Exposures:**

Hospitalization type (inpatient, for observation only), comorbidity burden, acute physiology score, and care directives.

**Main Outcomes and Measures:**

Mortality (inpatient, 30-day, and 30-day postdischarge), nonelective rehospitalization, and discharge disposition (home, home with home health assistance, regular skilled nursing facility, or custodial skilled nursing facility).

**Results:**

In total, 1 384 025 hospitalizations were identified, of which 1 155 034 (83.5%) were inpatient and 228 991 (16.5%) were for observation only. These hospitalizations involved 679 831 patients (mean [SD] age, 61.4 [18.1] years; 362 582 female [53.3%]). The number of for-observation-only hospitalizations increased from 16 497 (9.4%) in the first year of the study to 120 215 (20.5%) in the last period of the study, whereas inpatient hospitalizations with length of stay less than 24 hours decreased by 33% (from 12 008 [6.9%] to 27 108 [4.6%]). Illness burden measured using administrative data or acute physiology score increased significantly. The proportion of patients with a Comorbidity Point Score of 65 or higher increased from 20.5% (range across hospitals, 18.4%-26.4%) to 28.8% (range, 22.3%-33.0%), as did the proportion with a Charlson Comorbidity Index score of 4 or higher, which increased from 28.8% (range, 24.6%-35.0%) to 38.4% (range, 31.9%-43.4%). The proportion of patients at or near critical illness (Laboratory-based Acute Physiology Score [LAPS2] ≥110) increased by 21.4% (10.3% [range across hospitals, 7.4%-14.7%] to 12.5% [range across hospitals, 8.3%-16.6%]; *P* < .001), reflecting a steady increase of 0.07 (95% CI, 0.04-0.10) LAPS2 points per month. Unadjusted inpatient mortality in the first year of the study was 2.78% and in the last year was 2.71%; the corresponding numbers for 30-day mortality were 5.88% and 6.15%, for 30-day postdischarge mortality were 3.94% and 4.22%, and for nonelective rehospitalization were 12.00% and 12.81%, respectively. All outcomes improved after risk adjustment. Compared with the first month, the final observed to expected ratio was 0.79 (95% CI, 0.73-0.84) for inpatient mortality, 0.86 (95% CI, 0.82-0.89) for 30-day mortality, 0.90 (95% CI, 0.85-0.95) for 30-day nonelective rehospitalization, and 0.87 (95% CI, 0.83-0.92) for 30-day postdischarge mortality. The proportion of nonelective rehospitalizations meeting public reporting criteria decreased substantially over the study period (from 58.0% in 2010-2011 to 45.2% in 2017); most of this decrease was associated with the exclusion of observation stays.

**Conclusions and Relevance:**

This study found that in this integrated system, the hospitalization rate decreased and risk-adjusted hospital outcomes improved steadily over the 7.5-year study period despite worsening case mix. The comprehensive results suggest that future assessments of care quality should consider the implications of numerator and denominator definitions, display multiple metrics concurrently, and include all hospitalization types and detailed data.

## Key Points

**Question:**

How did hospitalization outcomes, including rehospitalization, change in an integrated health care delivery system between 2010 and 2017?

**Findings:**

In this cohort study of 1 384 025 hospitalizations among 679 831 patients in 1 integrated health care delivery system, the hospitalization rate; adjusted inpatient, 30-day, and 30-day postdischarge mortality rates; and nonelective rehospitalization rates decreased despite worsening case mix. The proportion of hospitalizations subject to public reporting also decreased, primarily owing to increases in for-observation-only hospitalizations.

**Meaning:**

Findings of this study suggest that hospitalizations, rehospitalizations, and mortality rates can be reduced simultaneously, but a single measure is unlikely to accurately describe these changes.

## Teaser

This cohort study examines rehospitalization factors, hospitalization and mortality, discharge disposition, and the changes in these variables over time among all adult patients in the Kaiser Permanente Northern California health system.

## Introduction

After the 2009 publication of the article by Jencks et al,^1^ the Centers for Medicare & Medicaid Services (CMS) introduced the rehospitalization metric as a publicly reported quality measure, which was followed by the Hospital Readmissions Reduction Program (HRRP).^2^ The HRRP imposed penalties (beginning on October 1, 2012) to create incentives for improvement in care coordination among fee-for-service Medicare members. Among health systems that provide care for Medicare Advantage members, the HRRP sanctions play a smaller role. However, health plans have strong incentives to decrease readmissions as part of the CMS Five-Star Quality Rating System.^3^ Hospitals with excess readmissions can experience a decrease in their rating that is, in turn, associated with substantial reductions in reimbursement and their ability to enroll new members. The HRRP and the Five-Star Quality Rating System were instituted at the same time. Consequently, considerable research and regulations have focused on rehospitalization for community-acquired pneumonia, acute myocardial infarction, and congestive heart failure, the 3 original targeted conditions that remain the program’s centerpiece.

The consensus in the United States is that rehospitalization rates have decreased, which has raised 3 major quality concerns. First, are decreases in 30-day rehospitalization rates accompanied by increases in inpatient, 30-day, and/or 30-day postdischarge mortality rates?^4,5^ Second, is the exclusion of patients admitted for observation only (a CMS mandate, whose value has been questioned,^6,7^ based on a physician’s assessment to keep a patient in the hospital for 2 midnights) falsely lowering the true rehospitalization rates and precluding adequate quality assessment?^8,9,10,11^ Third, in an era in which the hospitals and health care systems in the United States have transitioned to electronic medical records (EMRs) that can capture detailed clinical data, why does public reporting still rely on administrative data, despite calls for better risk adjustment?^12,13^

One issue that has received limited attention is the population hospitalization rate. Using 2008 Medicare data on patients with community-acquired pneumonia, acute myocardial infarction, and congestive heart failure, Epstein et al^14^ highlighted the strong association between population hospitalization rates and rehospitalization rates. Subsequently, using Medicare data from 2008 to 2013, Dharmarajan et al^15^ showed a parallel reduction in both hospitalization and 30-day rehospitalization rates despite patients’ worsening comorbidity burden. This decrease was not associated with increases in a composite outcome (nonelective rehospitalization or death within 30 days of discharge).

A potential problem with the current literature on rehospitalization is that it can lead to a fragmented view, given that each study addresses discrete issues. In this cohort study, we took a broad perspective to examine hospitalization and rehospitalization. Using clinically intuitive definitions, we considered how multiple factors described in recent literature have played out in 1 integrated health care delivery system. We sought to identify and understand several factors associated with the rehospitalization metric, including outcome definitions, illness burden measures, and criteria to define denominators and numerators. Recent literature suggests that several of these factors could be trending in different directions, making it difficult to discern the association between the rehospitalization metric and actual quality of care.

Using detailed clinical data from a contemporary cohort in a health system that maximizes the use of EMRs, we expanded on recent research by examining outcomes in all adults. We evaluated the changes in hospitalization and metric definitions in the period before and after the implementation of the HRRP penalty phase. We reported on both rehospitalization and survival before and after discharge and considered illness burden (including acute physiology, not just comorbidities), the population hospitalization rate, discharge disposition, and changes in these factors over time. The study setting was Kaiser Permanente Northern California (KPNC), an integrated health care delivery system with 21 hospitals that serve patients with Medicare Advantage, Medicaid, and Kaiser Foundation Health Plans. Robust KPNC database systems provided us with an opportunity to examine hospitalization outcomes more broadly and to quantify temporal trends for factors that have not been extensively described in the existing literature.

## Methods

This cohort study was approved by the KPNC Institutional Review Board for the Protection of Human Subjects, which waived the requirement for informed consent from participants given that this study involved only data. The study followed the Strengthening the Reporting of Observational Studies in Epidemiology (STROBE) reporting guideline.

Under a mutual exclusivity agreement, 9500 salaried physicians of The Permanente Medical Group provide care for 4.3 million members of the Kaiser Foundation Health Plan (KFHP) at facilities owned by Kaiser Foundation Hospitals. Deployment of a systemwide EMR (Epic) was completed in 2010.

Within KPNC, a 21-hospital system that has been described elsewhere,^16,17,18,19,20^ and using its EMR system, we identified hospital stays for patients who met these criteria: hospital discharge from June 1, 2010, through December 31, 2017 (excluding nonovernight 1-day surgical procedures); age 18 years or older at hospitalization; and hospitalization that was not for childbirth (stays for postdelivery complications were included). We linked hospital stays for transferred patients and identified all deaths using public and internal sources.^16,17,18,19,20^ We assigned inpatient and 30-day mortality rates to the admitting hospital and 30-day rehospitalization and mortality rates to the discharging hospital. Using the data systems and study methods, we classified all hospitalizations as an index hospitalization, a rehospitalization, or both.

Given KPNC practice standards that discourage direct admission to the hospital from the outpatient clinic setting, we considered rehospitalizations as nonelective if they began in the emergency department, if the principal diagnosis was an ambulatory care–sensitive condition,^21^ or if they began in an outpatient clinic and the patient had elevated severity of illness (mortality risk of 7.2% based on acute physiology score alone, described later).^20^ Hospitalizations that did not meet the nonelective criteria were also considered elective. We examined hospitalized patients’ KFHP membership data to ascertain whether a discharge satisfied the criteria of the National Committee for Quality Assurance’s Healthcare Effectiveness Data and Information Set (HEDIS). These HEDIS criteria stipulate continuous health plan membership in the 12 months before and the 30 days after hospital discharge, with a maximum gap in coverage of 45 days in the preceding 12 months.^22,23^ In addition, we captured whether a hospitalization was *inpatient* or *for observation only*. If a hospitalization began as for observation only but transitioned to inpatient status, we classified it as inpatient. To calculate hospitalization rates among KFHP members, we obtained monthly membership counts from internal reports.

The dependent variables were hospitalization, inpatient mortality, 30-day mortality, nonelective rehospitalization within 30 days of hospital discharge, death within 30 days of hospital discharge, and a composite outcome (nonelective rehospitalization and/or death within 30 days of hospital discharge).

We also captured KFHP membership, long-term comorbidity burden, severity of illness, and code status.^19^ At KPNC, each month, all adults with a medical record number are assigned a Comorbidity Point Score, version 2 (COPS2), which is based on CMS Hierarchical Condition Categories (score range, 0-1014 [scores above 300 are rare], with higher scores indicating increased mortality risk). Based on the experience with KPNC’s early warning system, now operational in all 21 hospitals,^24,25,26^ inpatients with a COPS2 of 65 or higher are evaluated by palliative care teams (a score above this threshold is associated with a high risk of in-hospital deterioration). For comparison, we assigned each hospitalization a Charlson Comorbidity Index score (range, 0-24 [scores above 5 are uncommon], with higher scores indicating increased mortality risk), using the methods of Deyo et al.^27^ At KPNC, patients are assigned a Laboratory-based Acute Physiology Score, version 2 (LAPS2; range: 0-414 [scores above 200 are uncommon] on admission and every hour after hospitalization, with higher scores indicating worsening instability), including a score assigned at 0800 on the discharge day (LAPS2dc). For example, in July 2018, the median hourly LAPS2 among all patients in the intensive care unit was 110, whereas the median ward score was 52. It is not possible to admit a patient to KPNC hospitals without specifying code status, which can be subsequently updated. We classified each patient’s care directive as full code or not (which included partial code, do not resuscitate, and comfort care only).^19^

We also captured age at hospitalization, sex, hospitalization venue (emergency department or not), total index hospital length of stay (LOS), whether a patient experienced any overnight hospitalization in the first 7 days and separately in the 8 to 30 days before the index hospitalization,^20^ discharge disposition (home, regular or custodial skilled nursing facility [SNF], and home health services), and referral to hospice. We combined the Healthcare Cost Utilization Project’s single-level Clinical Classification Software diagnosis categories to categorize all *International Classification of Diseases, Ninth Revision *and *International Statistical Classification of Diseases and Related Health Problems, Tenth Revision (ICD-10)*, hospitalization or principal diagnosis codes into 30 groups called Primary Conditions.^19,20^

We used 3 cohort definitions: all (ALL), inpatient (INP), and subject to public reporting (PUB). The ALL cohort included all hospitalizations or discharges in the denominator. The INP cohort included only inpatient hospitalizations in the denominator. The PUB cohort approximated the HEDIS construct used in the CMS Five-Star Quality Rating System,^3^ which does not include mortality as an outcome. The PUB cohort included only discharges in which patients met the HEDIS membership requirements and was restricted to inpatient hospitalizations in both the numerator and denominator. Additionally, the PUB cohort excluded inpatient stays of less than 24 hours and for-observation-only hospitalizations, inpatient rehospitalizations without a preceding inpatient hospitalization within 30 days (ie, rehospitalizations following for-observation-only hospitalization), and inpatient and postdischarge deaths unless they occurred as part of an eligible inpatient rehospitalization.

### Statistical Analysis

For all risk-adjusted analyses, we developed statistical models using data from the first year (June 1, 2010, to May 31, 2011) as a baseline. We used logistic regression with variables based on models that were developed and tested earlier and described in previous reports^16,19,20^: age, sex, COPS2, admission LAPS2, index discharge LOS, previous hospitalizations, discharge code status, diagnosis (Primary Condition), and KFHP membership status at the time of discharge. For the first 2 cohorts (ALL and INP), we developed risk adjustment models for all outcomes. For the PUB cohort, for which mortality was not a reported outcome, we only modeled rehospitalization. We used these models to estimate the probability of observing the outcome (expected) and the observed to expected ratios for multiple subgroups of patients and across time.

For the graphical displays of patient outcomes and observed to expected ratios, we used a 3-month moving average technique and estimated the monthly observed and expected outcomes as a mean of the past, present, and future months (thus, the May 2015 data point was based on index hospitalizations from April 1, 2015, through June 30, 2015). We deseasonalized moving averages using the X-11 procedure.^28^ We assessed monthly trends through regression models with an autocorrelated error structure^29^ and differences in proportions with the χ^2^ test using 2-tailed, unpaired tests. Two-sided *P* < .05 was considered statistically significant. All statistical calculations and plots were performed with SAS, version 9.4 (SAS Institute Inc).

For reference purposes in the graphical displays, we divided the period before the implementation of the HRRP penalties into 2 epochs: (1) reference year for multivariate analyses (June 1, 2010, to May 31, 2011) and (2) time until the beginning of the HRRP penalty phase (June 1, 2011, to September 30, 2012). We divided the period after the implementation of HRRP penalties into 2 epochs: (1) period that matches the dates used by Gupta et al^4^ (October 1, 2012, to December 31, 2014) and (2) the remaining years (January 1, 2015, to December 31, 2017).

## Results

We identified 1 384 025 hospitalizations among 679 831 patients (mean [SD] age, 61.4 [18.1] years; 362 582 female [53.3%]). Of the total hospitalizations, 1 155 034 (83.5%) were inpatient and 228 991 (16.5%) were for observation only. Table 1 summarizes cohort characteristics before and after the implementation of the HRRP statutory financial penalties. The number of for-observation-only hospitalizations increased from 16 497 (9.4%) in the first year of the study (June 1, 2010, to May 31, 2011) to 120 215 (20.5%) in the last study period after penalty implementation (January 1, 2015, to December 31, 2017), whereas inpatient hospitalizations with LOS of less than 24 hours decreased by 33% (from 12 008 [6.9%] to 27 108 [4.6%]) between these periods. These changes were more pronounced among patients with acute myocardial infarction, community-acquired pneumonia, and congestive heart failure, in whom the absolute numbers also increased (eAppendix 1 in the Supplement). The proportion of hospitalizations in which patients met the strict HEDIS membership definition decreased slightly (81.8% to 78.9%). In contrast, the proportion of hospitalizations that met the public reporting definition (which excluded for-observation-only and short inpatient LOS) decreased by 14.5% from 68.6% (range across hospitals, 59.7%-76.2%) to 58.7% (range across hospitals, 40.0%-68.9%).

**Table 1.** Cohort Characteristics^a^

Variable	Proportion (Range), %				
	Before HRRP Penalty Phase Implementation		After HRRP Penalty Phase Implementation		Total
	June 2010-May 2011	June 2011-September 2012	October 2012-December 2014	January 2015-December 2017	
Hospitalizations, No.	175 284	230 783	390 550	587 408	1 384 025
All patients, No.	127 384	161 311	249 955	350 628	679 831
Inpatient^b^ status	90.6 (82.0-95.6)	87.1 (77.1-94.1)	84.0 (71.8-91.1)	79.5 (58.2-89.5)	83.5 (71.2-90.5)
For-observation-only status	9.4 (4.4-18.0)	12.9 (5.9-22.9)	16.0 (8.9-28.2)	20.5 (10.5-41.8)	16.5 (9.5-28.8)
Inpatient stay <24 h	6.9 (4.3-9.3)	6.0 (4.0-7.7)	4.8 (3.1-6.6)	4.6 (1.9-6.8)	5.2 (3.5-6.8)
Transported in^c^	4.7 (1.2-8.4)	4.6 (1.6-8.8)	4.3 (2.1-9.0)	4.4 (0.3-8.7)	4.5 (1.5-8.7)
Mean age, y	64.5 (60.4-69.3)	64.6 (60.3-68.9)	65.3 (61.8-69.7)	65.6 (62.7-70.2)	65.2 (62.0-69.7)
Male	45.9 (41.0-53.1)	46.3 (40.7-53.2)	47.1 (42.0-53.9)	48.2 (44.3-53.7)	47.3 (42.7-53.6)
KFHP membership	94.7 (86.4-97.8)	94.7 (84.5-98.0)	94.2 (84.6-98.1)	92.7 (65.0-97.7)	93.7 (77.4-97.9)
Met strict HEDIS membership definition^d^	81.8 (74.2-85.3)	82.2 (72.5-86.6)	80.9 (71.4-85.7)	78.9 (54.2-84.7)	80.4 (65.2-85.1)
Met public reporting definition^d^	68.6 (59.7-76.2)	66.9 (55.7-76.2)	64.1 (52.4-71.9)	58.7 (40.0-68.9)	62.8 (48.6-70.2)
Hospitalization via ED	66.7 (52.0-85.6)	67.2 (55.5-80.3)	69.1 (56.4-81.8)	71.6 (56.3-86.3)	69.6 (56.2-81.3)
CCI score, median^e^	2.0 (1.0-2.0)	2.0 (1.0-2.0)	2.0 (1.0-3.0)	2.0 (2.0-3.0)	2.0 (2.0-2.0)
≥4	28.8 (24.6-35.0)	30.4 (25.0-37.6)	33.3 (27.4-40.4)	38.4 (31.9-43.4)	34.4 (28.7-40.0)
COPS2, mean^f^	37.3 (34.8-44.5)	40.0 (35.6-47.1)	45.3 (39.2-54.3)	47.9 (39.7-53.4)	44.5 (38.7-51.6)
≥65	20.5 (18.4-26.4)	22.5 (18.8-28.1)	26.7 (21.8-33.7)	28.8 (22.3-33.0)	26.1 (21.2-31.5)
LAPS2^f^					
Admission	55.3 (46.4-64.0)	55.7 (46.4-64.4)	57.7 (47.0-67.3)	59.8 (48.9-69.5)	58.0 (47.6-66.8)
Discharge	46.3 (41.1-51.7)	46.4 (43.0-51.1)	46.1 (42.5-51.2)	46.8 (41.8-51.3)	46.5 (42.4-50.6)
≥110	10.3 (7.4-14.7)	10.8 (7.1-15.1)	11.7 (6.9-15.8)	12.5 (8.3-16.6)	11.7 (7.6-15.5)
Full code at discharge	85.1 (77.8-89.8)	84.9 (77.7-91.7)	84.2 (77.7-90.6)	84.3 (77.0-90.4)	84.5 (77.4-90.6)
Mean LOS, d	5.0 (4.2-5.7)	5.0 (4.0-5.6)	4.9 (3.8-5.5)	4.8 (3.9-5.5)	4.9 (3.9-5.4)
Discharge disposition^g^					
Regular discharge	75.9 (71.6-85.0)	74.4 (69.1-85.0)	72.5 (65.3-84.8)	71.8 (53.1-87.5)	72.9 (62.2-86.1)
Home health	12.3 (7.1-18.5)	13.4 (7.4-19.4)	15.9 (7.5-23.8)	17.8 (6.4-31.1)	15.8 (6.9-23.2)
SNF					
Regular	10.2 (6.8-15.9)	10.7 (6.3-15.4)	10.1 (6.5-15.9)	9.3 (5.3-13.6)	9.8 (5.9-14.7)
Custodial	1.6 (0.8-2.6)	1.6 (0.9-3.1)	1.5 (0.8-2.8)	1.2 (0.5-2.3)	1.4 (0.8-2.6)
Hospice referral	2.5 (1.8-3.8)	2.5 (1.7-4.5)	2.6 (1.8-5.1)	2.6 (1.6-4.2)	2.6 (1.7-4.4)
Outcomes^h^					
Mortality					
Inpatient	2.8 (1.9-3.8)	2.7 (1.9-3.8)	2.8 (1.9-3.5)	2.8 (2.0-3.3)	2.8 (2.1-3.3)
30-d	5.9 (3.8-7.4)	5.9 (4.0-7.4)	6.1 (4.1-7.7)	6.1 (4.2 7.6)	6.1 (4.1 7.5)
Rehospitalization					
Any	14.4 (12.4-16.7)	14.3 (12.1-17.2)	14.1 (11.5-17.4)	14.6 (12.8-17.1)	14.4 (12.4-17.1)
Any nonelective	12.0 (9.9-14.8)	12.0 (9.7-15.2)	12.0 (10.0-15.5)	12.6 (10.3-15.4)	12.2 (10.2-15.3)
Nonelective inpatient	11.1 (9.0-13.9)	10.6 (7.8-13.7)	10.2 (7.7-12.7)	10.3 (8.1-12.6)	10.4 (8.1-12.6)
Nonelective observation	1.2 (0.7-1.8)	1.9 (0.6-3.1)	2.3 (1.1-3.6)	2.9 (1.7-5.3)	2.3 (1.3-3.5)
30-d Postdischarge mortality	3.9 (2.4-5.5)	4.0 (2.9-5.3)	4.1 (2.8-5.5)	4.1 (2.7-5.3)	4.1 (2.7-5.4)
Composite outcome	14.8 (11.6-18.8)	14.8 (12.6-18.8)	14.9 (13.0-19.1)	15.5 (13.1-18.8)	15.1 (12.9-18.9)

Abbreviations: CCI, Charlson Comorbidity Index; COPS2, Comorbidity Point Score, version 2; ED, emergency department; HEDIS, Healthcare Effectiveness Data and Information Set; HRRP, Hospital Readmissions Reduction Program; KFHP, Kaiser Foundation Health Plan; LAPS2, Laboratory-based Acute Physiology Score, version 2; LOS, length of stay; SNF, skilled nursing facility.

^a^ Rates used hospitalization episodes (which can include linked stays for patients who were transported) as the denominator. Numbers in parentheses refer to the range across the 21 study hospitals. Time before the penalty phase was divided into 2 epochs: (1) reference period for multivariate analyses and other comparisons and (2) time until the HRRP penalty phase began. Time after the penalty phase was divided into 2 epochs: (1) period matching that of the study by Gupta et al^4^ and (2) period covering the remaining years.

^b^ Hospital episodes in which patients transitioned from for-observation-only to inpatient status were classified as inpatient.

^c^ Refers to patients whose linked hospitalization episode began at a hospital not owned by Kaiser Foundation Hospitals. These hospitalizations had elevated inpatient and 30-day mortality compared with the rest of the Kaiser Permanente Northern California cohort. See Escobar et al^16,19,20^ for details.

^d^ The HEDIS membership definition restricted the denominator to patients with continuous health plan membership in the 12 months before and the 30 days after hospital discharge, with a maximum gap in coverage of 45 days in the preceding 12 months. The public reporting definition included only patients who met the HEDIS membership criteria and excluded observation and inpatient hospitalizations with LOS less than 24 hours.

^e^ Calculated using the methods of Deyo et al.^27^

^f^ The COPS2 was assigned on the basis of all diagnoses incurred by a patient in the 12 months before the index hospitalization. The univariate associations of COPS2 with 30-day mortality were as follows: 0 to 39, 1.7%; 40 to 64, 5.2%; 65 or greater, 9.0%. LAPS2 was assigned based on a patient’s worst vital signs, pulse oximetry, neurological status, and 16 laboratory test results in the preceding 24 (discharge LAPS2) or 72 hours (admission LAPS2). The univariate associations of an admission LAPS2 with 30-day mortality were as follows: 0 to 59, 1.0%; 60 to 109, 5.0%; 110 or greater, 13.7%; for LAPS2dc, the association is 0 to 59, 2.2%; 60 to 109, 8.1%; 110 or greater, 20.5%.

^g^ Disposition among patients discharged alive from the hospital. Hospice referral was independent of discharge disposition.

^h^ Transported-in patients were excluded from inpatient and 30-day mortality data. Only patients who survived to discharge were included in the postdischarge outcomes. Nonelective rehospitalizations were those that began in the ED, were for an ambulatory care sensitive condition, and/or had a LAPS2 60 or greater, as described in Escobar et al.^20^ Composite outcome was nonelective rehospitalization or death within 30 days after discharge.

The overall comorbidity burden, as measured by the COPS2, increased steadily by 0.16 (95% CI, 0.13-0.20) points per month. Table 1 shows that the proportion of patients with a COPS2 of 65 or higher increased from 20.5% (range across hospitals, 18.4%-26.4%) to 28.8% (range across hospitals, 22.3%-33.0%; *P* < .001), as did the proportion with a Charlson Comorbidity Index score of 4 or higher, which increased from 28.8% (range across hospitals, 24.6%-35.0%) to 38.4% (range across hospitals, 31.9%-43.4%; *P* < .001). Acute physiology scores also increased steadily each month, as did the proportion of patients at or near critical illness (LAPS2 ≥110), which increased by 21.4% (from 10.3% [range across hospitals, 7.4%-14.7%] to 12.5% [range across hospitals, 8.3%-16.6%], reflecting a steady increase of 0.07 [95% CI, 0.04-0.10] LAPS2 points per month; *P* < .001), although this increase was not statistically significant among patients with pneumonia (where the proportion fell from 19.1% to 17.5%; *P* = .10) or congestive heart failure (20.0% to 19.8%; *P* = .23) (eAppendix 1 in the Supplement). The proportion of patients with acute myocardial infarction at or near critical illness decreased from 14.5% (range across hospitals, 5.4%-24.0%) to 12.6% (range across hospitals, 8.0%-37.5%; *P* < .001) (eAppendix 1 in the Supplement).

Despite increased acuity and comorbidity, hospital LOS remained stable, as did the proportion of patients discharged alive as full code. The proportion of patients discharged with home health services increased from 12.3% (range across hospitals, 7.1%-18.5%) to 17.8% (range across hospitals, 6.4%-31.1%]). Discharges to other venues decreased: regular discharge home, from 75.9% (range across hospitals, 71.6%-85.0%) to 71.8% (range across hospitals, 53.1%-87.5%); regular SNF, from 10.2% (range across hospitals, 6.8%-15.9%) to 9.3% (range across hospitals, 5.3%-13.6%); and custodial SNF, from 1.6% (range across hospitals, 0.8%-2.6%) to 1.2% (range across hospitals, 0.5%-2.3%). Table 1 also shows that, with the exception of nonelective rehospitalizations that were for observation only (which more than doubled from 1.2% [range across hospitals, 0.7%-1.8%] to 2.9% [range across hospitals, 1.7%-5.3%]), unadjusted outcomes remained constant over the study period.

**Figure 1.**
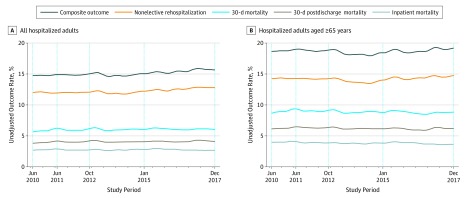
Unadjusted Outcome Rates Among All Hospitalized Adults and Hospitalized Adults Aged 65 Years or Older The trends for unadjusted outcomes were either not statistically significant or, if significant, trivial (eg, the trend for 30-day postdischarge mortality in patients aged 65 years or older was −0.002% [*P* = .007]). Relative constancy of unadjusted rates was similar for various patient subsets (eg, those with community-acquired pneumonia; see eAppendix 2 in the Supplement for these additional figures). The study period comprised the baseline period (June 2010 to May 2011), beginning of Hospital Readmissions Reduction Program penalty phase (October 2012), and end of study period (December 2017). This latter period is split into 2 periods based on the study by Gupta et al^4^ (October 2012 to December 2014).

Figure 1 shows unadjusted rates for 5 outcomes among all hospitalizations (left panel) and hospitalizations for patients aged 65 years or older (right panel). Among all patients, inpatient mortality (first year, 2.78%; last year, 2.71%), 30-day mortality (first year, 5.88%; last year, 6.15%), 30-day postdischarge mortality (first year, 3.94%; last year, 4.22%), nonelective rehospitalization (first year, 12.00%; last year, 12.81%), and composite outcome (first year, 14.77%; last year, 15.76%) rates remained relatively constant over the study period. Among patients aged 65 years or older, inpatient mortality (first year, 4.05%; last year, 3.69), 30-day mortality (first year, 9.00%, last year, 8.84%); 30-day postdischarge mortality (first year, 6.25%, last year, 6.32%); nonelective rehospitalization (first year, 14.23%, last year, 14.64%), and composite outcome (first year, 18.71%, last year, 19.13%) rates also remained relatively constant over the study period.

**Figure 2.**
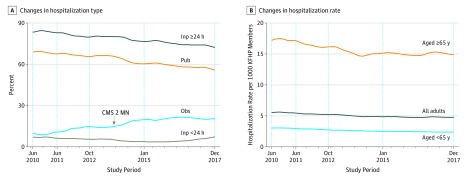
Changes in Hospitalization Type and Hospitalization Rate Over Time The study period comprised the baseline period (June 2010 to May 2011), beginning of Hospital Readmissions Reduction Program penalty phase (October 2012), and end of study period (December 2017). This latter period is split into 2 periods based on the study by Gupta et al^4^ (October 2012 to December 2014). CMS 2 MN indicates the promulgation date of the Centers for Medicare & Medicaid Services’ 2-midnight rule; Inp ≥24 h/<24 h, 24 hours or more/less inpatient length of stay; KFHP, Kaiser Foundation Health Plan; Obs, observation; and Pub, meeting public reporting specifications.

Figure 2 displays time trends for hospitalization type and hospitalization rate. The proportion of patients with short (which went from 6.9% in the first year to 6.1% in the last year) or observation (which went from 9.4% in the first year to 20.4% in the last year) hospitalizations increased (left panel), but this increase was not associated with increased hospitalization rates (right panel). These results were similar among patients of all ages (eAppendix 3 in the Supplement). The population hospitalization rate decreased by 13% among all adults (from 5.5 to 4.8 discharges per thousand KFHP members; *P* < .001), by 20% (from 3.0 to 2.4 per thousand KFHP members; *P* < .001) for patients younger than 65 years, and by 13% (17.2 to 14.9 per thousand KFHP members; *P* < .001) for patients aged 65 years or older.

**Figure 3.**
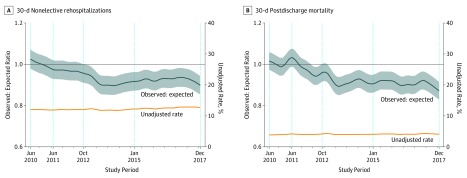
Adjusted and Unadjusted Rates of 30-Day Nonelective Rehospitalization and 30-Day Postdischarge Mortality By the final month of the study, the observed to expected ratio for 30-day nonelective rehospitalization was 0.90 (95% CI, 0.85-0.95) and for 30-day postdischarge mortality was 0.87 (95% CI, 0.83-0.92). eAppendix 4 in the Supplement provides graphics for inpatient mortality (final observed to expected ratio, 0.79; 95% CI, 0.73-0.84), 30-day mortality (0.86; 95% CI, 0.82-0.89), and 30-day composite outcome (nonelective rehospitalization or death within 30 days of discharge, 0.90; 95% CI, 0.86-0.94). The study period comprised the baseline period (June 2010 to May 2011), beginning of Hospital Readmissions Reduction Program (HRRP) penalty phase (October 2012), and end of study period (December 2017). This latter period is split into 2 periods based on the study by Gupta et al^4^ (October 2012 to December 2014).

Figure 3 contrasts the unadjusted rate with the observed to expected ratio for nonelective rehospitalization (left panel) and for 30-day postdischarge mortality (right panel). Compared with the first year, the final observed to expected ratio was 0.90 (95% CI, 0.85-0.95) for 30-day nonelective rehospitalization and 0.87 (95% CI, 0.83-0.92) for 30-day postdischarge mortality. Similar decreases in adjusted outcome rates were seen for inpatient mortality (final observed rate was 0.79 [95% CI, 0.73-0.84]), 30-day mortality (0.86 [95% CI, 0.82-0.89]), and 30-day composite outcome (0.90 [95% CI, 0.86-0.94]) (eAppendix 2 in the Supplement). These reductions suggest that the system was able to maintain the same observed outcome rates despite worsening case mix.

Table 2 and eAppendix 5 in the Supplement contrast data from the first to the last years of the study, highlighting the association between denominator definitions and the proportions of all hospitalizations and rehospitalizations included. For example, the restricted inpatient denominator captured 90.6% of all hospitalizations in the first year of the study; by 2017, this proportion decreased to only 79.6%. Decreased capture was similar among patients with low COPS2 scores (90.3% vs 79.4%) or LAPS2 scores (90.0% vs 78.3%). This decrease was most pronounced in the restricted public reporting denominator. For example, in the 2010 to 2011 period, only 58.0% of nonelective rehospitalizations were captured, and this proportion decreased to 45.2% by 2017. In addition, Table 2 shows that, because the public reporting metric excludes out-of-hospital deaths, by 2017 only 2.2% of inpatient deaths, 3.4% of 30-day deaths, and 5.0% of 30-day postdischarge deaths were captured by public reporting.

**Table 2.** Association of Denominator Definition With Outcome Capture

Outcome	Denominator, %^a^			
	Inpatient Hospitalization		Publicly Reported Hospitalization	
	June 2010-May 2011	January 2017-December 2017	June 2010-May 2011	January 2017-December 2017
Hospitalization				
Any	90.6	79.6	68.6	57.2
With COPS2				
≥65^b^	91.8	80.3	69.4	61.3
<65	90.3	79.4	68.4	55.5
With LAPS2				
≥110^b^	96.2	89.2	57.5	52.4
<110	90.0	78.3	69.8	57.9
Death				
Inpatient	98.1	93.6	4.3	2.2
30-d	96.5	90.2	5.6	3.4
Nonelective rehospitalization^c^	92.1	81.8	58.0	45.2
Death within 30 d of discharge	95.3	88.0	7.4	5.0
Composite outcome^d^	92.8	83.1	48.5	37.6

Abbreviations: COPS2, Comorbidity Point Score, version 2; LAPS2, Laboratory-based Acute Physiology Score, version 2.

^a^ Shown are the proportions of outcomes captured when the denominator was restricted to only inpatient or publicly reported hospitalizations. The public reporting definition restricted the denominator to patients with continuous health plan membership in the 12 months before and the 30 days after hospital discharge, with a maximum gap in coverage of 45 days in the preceding 12 months. Excluded from both the numerator and denominator were for-observation-only hospitalizations and inpatient hospitalizations with length of stay less than 24 hours as well as mortality as an outcome.

^b^ For a description of the COPS2 and LAPS2 scores, see text, Table 1 notes, and Escobar et al.^19^ Patients with scores at these levels were very ill.

^c^ For the definition of nonelective rehospitalization, see text and Escobar et al.^20^ Hospitalizations for observation were included in the base total.

^d^ The composite outcome was nonelective rehospitalization or death within 30 days of hospital discharge. Hospitalizations for observation were included.

## Discussion

Using data from a large contemporary cohort of all adult patients in the integrated KPNC system, this cohort study quantified improvements in 30-day hospital outcomes. Although hospitalized patients appeared to be sicker on admission over time, as evidenced by traditional measures such as the Charlson Comorbidity Index score, automated long-term scores (COPS2), and automated acute physiology scores (LAPS2), the outcomes improved for these patients. Hospitalization, rehospitalization, and mortality rates decreased simultaneously among all adults, not just those eligible for Medicare, Medicaid, or Kaiser Foundation Health Plans.

These results complement and expand on the work of Epstein et al^14^ and Dharmarajan et al,^15^ highlighting that reducing hospitalization, rehospitalization, and mortality rates simultaneously is possible. Further evidence of the changing nature of hospitalization was the increasing proportion of patients discharged with home health services, which is most likely associated with changes in practice that emphasize home care. The proportion of hospitalizations subject to public reporting decreased substantially over the study period. Most of this decrease was associated with the increased rate of for-observation-only hospitalization.

In addition to highlighting the gap between what public reporting metrics capture and what is happening in KPNC hospitals, the results of this study also illustrate the growing gap between data used by regulatory entities and data that are becoming widely used outside the research and regulatory settings. Although KPNC may be unusual in assigning automated severity of illness,^19^ inpatient deterioration risk,^24,25,26^ and rehospitalization risk^20^ scores to all of its adult patients, increasing numbers of hospitals are deploying automated early warning systems such as the electronic Cardiac Arrest Triage score^30^ or the Rothman Index,^31^ which demonstrates the increased availability of data that could have substantial implications for hospital rankings.^12,13^ Because most studies do not have data on severity of illness, comparing our findings is difficult. One notable exception was the study by Gupta et al,^4^ which found increased mortality where we did not but did report some admission physiological data (which, in contrast to the data in this study, remained stable before and after the HRRP penalties were implemented).

Current reporting schema offer an incomplete, and certainly not patient-centered, perspective on the quality of hospital and postdischarge care. Because of the financial penalties associated with the rehospitalization metric, hospitals devote enormous resources to preventing rehospitalizations. In the absence of composite views displaying multiple metrics and balancing measures, however, it is difficult to assess whether these efforts are the most appropriate way to improve quality or whether higher scores in a single metric are necessarily associated with improved quality.

These findings also suggest potentially fruitful areas for future research. Given that regulations encourage the use of 2 types of hospitalization (inpatient and for observation only), expanding on the work of researchers, such as Nuckols et al^8^ and Sabbatini et al,^9^ becomes important to better understand the characteristics of observation hospitalizations, including their difference from inpatient hospitalizations. Researchers must study the consequences of this shift on patients, and not just analyze its implications for hospital rankings, which may be minimal.^32^ Adverse consequences to patients could include increased out-of-pocket costs and differential access to care coordination. Future research should also include more granular analysis of the details of the discharge process and hospital-outpatient information transfer and referral. Another area that deserves continued attention is examination of the association between hospitalization and rehospitalization rates, which could vary dramatically depending on what incentives and quality safeguards are in place. Further research is also needed into the association between rehospitalization rates and inpatient, 30-day, 30-day postdischarge, and out-of-hospital mortality rates. At the least, public reporting should incorporate 2-dimensional plots that permit visualization of which hospitals are doing well on 2 metrics simultaneously.

In an era in which an increasing proportion of patients have multimorbidity, we must address 2 challenges that face the medical profession. The first is the limited value of using mortality as a quality measure.^33^ The second is the need for prevention of hospitalization, including renewed efforts to find alternatives to hospitalization; after all, the best way to avoid rehospitalization is to not be hospitalized in the first place.

### Limitations

This study has several limitations to the generalizability of the findings. First, the KPNC cohort consists of an insured population receiving care from a system with an unusually high degree of integration. This integration is manifest in the availability of services aimed at preventing hospitalization, such as chronic condition management programs, a central call center, and electronic patient portals. Furthermore, as a capitated system, KPNC does not receive incentives to hospitalize patients. This integration and incentive structure may explain KPNC’s ability to decrease all of the outcome measures simultaneously. This finding contrasts with the results in a recent study by Joynt Maddox et al,^34^ who reported that participation in the CMS Innovation Bundled Payments for Care Improvement initiative was not associated with decreases in rehospitalization or mortality among 492 participating hospitals.

Second, KPNC’s for-observation-only hospitalization rate was relatively high, although some investigators have reported similar rates at individual hospitals.^35,36^ Patients in the present study appeared to be sicker over time, as evident from both diagnosis-based measures (Charlson Comorbidity Index score, COPS2) and acute physiology scores (LAPS2). Dharmarajan et al^15^ have also reported increasing comorbidity burden among hospitalized patients, but they did not have access to severity-of-illness data. Given that the use of physiological severity-of-illness scores outside the intensive care unit is not common in other health systems, we were not able to quantify the full extent of this trend and how generalizable it is.

## Conclusions

This cohort study showed that hospitalizations, rehospitalizations, and mortality can be decreased simultaneously. A single measure is unlikely to accurately describe these changes. New data elements available from contemporary EMRs, such as severity-of-illness scores and patient care directives, should become part of public reporting.

## Notes

Supplement. **eAppendix 1.** Supplemental Tables: Cohort Description **eAppendix 2.** Unadjusted Outcomes **eAppendix 3.** Proportions of Different Hospitalization Types **eAppendix 4.** Adjusted Outcomes **eAppendix 5.** Impact of Denominator Definition on Outcome Capture
