# Supplementary material for: Multiyear Rehospitalization Rates and Hospital Outcomes in an Integrated Health Care System
Source: JAMA Netw Open. 2019 Dec 4;2(12):e1916769. doi: 10.1001/jamanetworkopen.2019.16769 (PMC6902762; doi:10.1001/jamanetworkopen.2019.16769)
Supplement: Supplement. — eAppendix 1. Supplemental Tables: Cohort Description eAppendix 2. Unadjusted Outcomes eAppendix 3. Proportions of Different Hospitalization Types eAppendix 4. Adjusted Outcomes eAppendix 5. Impact of Denominator Definition on Outcome Capture [file jamanetwopen-2-e1916769-s001.pdf]

Supplementary Online Content

Escobar GJ, Plimier C, Greene JD, Liu V, Kipnis P. Multiyear rehospitalization rates and hospital outcomes in an integrated health care system. *JAMA Netw Open.* 2019;2(12):e1916769. doi:10.1001/jamanetworkopen.2019.16769

eAppendix 1. Supplemental Tables: Cohort Description
eAppendix 2. Unadjusted Outcomes
eAppendix 3. Proportions of Different Hospitalization Types
eAppendix 4. Adjusted Outcomes
eAppendix 5. Impact of Denominator Definition on Outcome Capture

This supplementary material has been provided by the authors to give readers additional information about their work.

©2019 Escobar GJ et al. *JAMA Network Open*

eAppendix 1. Supplemental Tables: Cohort Description

**Table S1A-1: Patients with community-acquired pneumonia (CAP)[a]**					
TIME PERIOD	*6/10-5/11*	*6/11-9/12*	*10/12-12/14*	*1/15-12/17*	*TOTAL*
	Before HRRP penalty phase		***HRRP penalties in effect***		
N (hospitalizations)	4,379	3,859	5,813	9,894	23,945
N (patients)	4,129	3,680	5,413	9,221	21,438
Inpatient (INP) (%)[b]	92.9 (78.9 - 99.6)	85.5 (54.7 - 99.2)	79.9 (60.2 - 95.2)	74.2 (59.3 - 91.0)	80.9 (66.6 - 95.4)
Observation (OBS) (%)	7.1 (0.4 - 21.1)	14.5 (0.8 - 45.3)	20.1 (4.8 - 39.8)	25.8 (9.0 - 40.7)	19.1 (4.6 - 33.4)
Inpatient stay < 24 hours	3.7 (0.9 - 9.2)	4.5 (0.9 - 8.0)	3.8 (1.6 - 5.9)	2.1 (0.7 - 4.1)	3.2 (2.0 - 5.4)
Transport-in[c]	5.4 (0.9 - 11.3)	5.6 (1.3 - 14.0)	5.0 (0.6 - 17.9)	4.9 (1.2 - 11.6)	5.1 (1.2 - 11.5)
Age (mean)	71.8 (67.2 - 78.5)	72.7 (65.2 - 77.8)	73.5 (67.3 - 77.1)	73.0 (66.9 - 76.6)	72.8 (67.9 - 77.0)
Sex (% male)	47.6 (41.5 - 55.8)	48.5 (43.2 - 58.9)	47.4 (42.9 - 53.8)	47.9 (43.6 - 60.3)	47.8 (45.2 - 56.6)
Kaiser Foundation Health Plan, Inc. member (%)	95.8 (86.6 - 100)	95.2 (84.0 - 99.7)	95.3 (84.9 - 98.8)	94.2 (68.9 - 98.5)	94.9 (81.1 - 98.8)
Met strict HEDIS membership definition[d] (%)	80.4 (72.0 - 84.6)	81.1 (68.6 - 87.7)	80.2 (69.9 - 86.5)	79.3 (59.3 - 85.5)	80.0 (67.0 - 85.8)
Met public reporting definition[d] (%)	71.3 (59.3 - 82.0)	65.3 (38.4 - 82.5)	60.5 (40.2 - 75.5)	56.4 (46.9 - 69.5)	61.5 (49.8 - 72.4)
Hospitalization via emergency department (%)	92.1 (86.4 - 97.0)	93.0 (82.6 - 97.6)	94.7 (88.9 - 98.6)	95.2 (88.0 - 100)	94.2 (89.5 - 98.0)
Charlson score[e] (median)	3.0 (2.0 - 4.0)	3.0 (2.0 - 4.0)	3.0 (3.0 - 4.0)	4.0 (3.0 - 4.0)	3.0 (3.0 - 4.0)
Charlson score ≥ 4 (%)	39.4 (29.4 - 51.7)	39.8 (30.2 - 52.6)	46.1 (36.6 - 55.8)	51.1 (44.2 - 55.9)	45.9 (39.0 - 50.6)
COPS2[f] (mean)	51.4 (43.7 - 57.9)	55.6 (46.0 - 66.3)	66.4 (58.9 - 75.2)	66.4 (49.8 - 73.6)	61.9 (50.9 - 69.5)
COPS2 ≥ 65 (%)	32.5 (26.7 - 39.6)	35.6 (25.9 - 44.3)	44.1 (37.6 - 52.7)	44.5 (32.6 - 50.7)	40.8 (32.0 - 47.4)
Admission LAPS2[f] (mean)	82.9 (75.6 - 90.1)	81.0 (70.5 - 86.2)	80.9 (68.0 - 88.2)	81.3 (71.3 - 87.8)	81.4 (74.3 - 87.2)
Discharge LAPS2 (mean)	58.7 (54.5 - 66.3)	59.2 (53.4 - 66.9)	58.2 (53.1 - 64.1)	57.6 (52.0 - 61.4)	58.2 (54.5 - 62.0)
LAPS2 ≥ 110 (%)	19.1 (10.4 - 27.3)	18.1 (10.1 - 23.8)	17.4 (6.0 - 23.5)	17.5 (10.4 - 25.1)	17.9 (11.5 - 23.2)
Full code at discharge (%)	70.3 (57.6 - 78.5)	68.1 (56.1 - 89.8)	66.8 (55.5 - 80.0)	69.3 (57.0 - 84.1)	68.7 (58.7 - 82.8)
Length of stay (days) (mean)	5.3 (4.4 - 6.3)	5.0 (3.8 - 6.1)	4.9 (3.9 - 6.8)	4.7 (3.8 - 5.4)	4.9 (4.0 - 5.6)
Discharge disposition[g] (%)					
Regular Discharge (%)	73.7 (59.8 - 83.2)	72.3 (53.9 - 84.4)	68.0 (50.7 - 79.8)	68.8 (51.9 - 83.2)	70.1 (53.6 - 80.5)

©2019 Escobar GJ et al. *JAMA Network Open*

Table S1A-1: Patients with community-acquired pneumonia (CAP)[a]					
TIME PERIOD	*6/10-5/11*	*6/11-9/12*	*10/12-12/14*	*1/15-12/17*	*TOTAL*
	Before HRRP penalty phase		***HRRP penalties in effect***		
Home Health (%)	13.7 (6.9 - 29.1)	15.1 (5.4 - 32.4)	19.8 (8.3 - 37.0)	19.8 (8.0 - 34.9)	17.9 (9.1 - 32.8)
Regular SNF (%)	9.4 (4.6 - 19.1)	9.8 (3.9 - 15.3)	9.7 (3.6 - 18.3)	9.4 (5.8 - 16.1)	9.5 (6.1 - 17.0)
Custodial SNF (%)	3.1 (1.1 - 7.3)	2.8 (0.7 - 8.1)	2.5 (1.3 - 5.6)	2.0 (0.4 - 4.3)	2.5 (0.5 - 5.0)
Hospice referral (%)	4.1 (0.6 - 7.8)	4.2 (0.9 - 9.6)	4.5 (3.0 - 8.3)	4.0 (0.7 - 9.0)	4.2 (2.2 - 7.3)
Outcomes[h]					
Inpatient mortality (%)	4.3 (1.8 - 8.4)	4.3 (1.2 - 8.9)	3.8 (1.1 - 6.2)	3.3 (0.4 - 4.6)	3.8 (0.8 - 5.2)
30-day mortality (%)	9.8 (6.3 - 14.9)	9.6 (5.0 - 16.2)	9.8 (6.0 - 13.6)	8.7 (2.6 - 10.9)	9.3 (5.8 - 11.6)
Any rehospitalization (%)	16.0 (11.3 - 22.9)	15.6 (10.6 - 26.1)	17.8 (12.5 - 25.8)	17.3 (13.3 - 22.8)	16.9 (12.8 - 20.0)
Any non-elective rehospitalization (%)	15.0 (9.5 - 22.7)	14.3 (9.8 - 23.9)	16.9 (11.8 - 23.5)	16.3 (12.9 - 21.7)	15.9 (12.2 - 19.0)
Non-elective inpatient rehospitalization (%)	14.1 (8.3 - 21.4)	12.9 (9.1 - 20.5)	14.4 (9.1 - 21.7)	13.5 (9.0 - 17.8)	13.7 (9.4 - 16.3)
Non-elective observation rehospitalization (%)	1.4 (0.5 - 3.3)	1.8 (0.4 - 4.7)	3.2 (1.4 - 6.6)	3.6 (0.9 - 8.2)	2.8 (1.0 - 5.3)
30-day post-discharge mortality (%)	6.8 (4.2 - 10.8)	6.3 (2.9 - 10.6)	7.2 (4.6 - 10.0)	6.7 (2.9 - 9.0)	6.8 (5.5 - 8.3)
Composite outcome (%)	19.6 (13.5 - 27.3)	18.8 (15.1 - 27.3)	21.5 (15.4 - 27.6)	20.6 (15.8 - 25.7)	20.3 (16.6 - 23.8)

FOOTNOTES

a Rates employ hospitalization episodes (which can include linked stays for patients who were transported) as the denominator. Numbers in parentheses always refer to the range across the 21 study hospitals. The period preceding the penalty phase of the Hospital Readmissions Reductions Program (HRRP), which took effect on 10/1/12, is divided into two epochs, with the first of these constituting the reference period for multivariate analyses and other comparisons. The period after the penalty phase also is divided into two epochs, with the first of these (through the end of 2014) matching that of the study of Gupta et al. and the second the remaining years.

b Hospital episodes where patients transitioned from OBS to INP status are classified as INP.

c Refers to patients whose linked hospitalization episode began at a hospital not owned by Kaiser Foundation Hospitals, Inc. These hospitalizations, involving 1,192 patients, had elevated inpatient (4.3%) and 30-day (11.9%) mortality, compared to 3.8% and 9.3% in the rest of the KPNC CAP cohort. Since we lacked information on their initial illness severity (the most important component of our mortality risk adjustment model) they are not included in inpatient and 30-day mortality analyses but are included in rehospitalization analyses, where admission illness severity plays a smaller role. See Escobar et al., 2008, 2013 and 2015, for details.

d The HEDIS membership definition restricts the denominator to patients with continuous health plan membership in the 12 months preceding and the 30 days following hospital discharge, with a maximum gap in coverage of 45 days in the preceding 12 months. The public reporting definition only includes patients meeting HEDIS membership criteria and excludes OBS hospitalizations and INP hospitalizations with length of stay < 24 hours.

©2019 Escobar GJ et al. *JAMA Network Open*

e Charlson score was calculated using the methodology of Deyo et al. (1992).

f The COmorbidity Point Score, version 2 (COPS2) is assigned based on all diagnoses incurred by a patient in the 12 months preceding the index hospitalization. The univariate relationship of COPS2 with 30-day mortality is as follows: 0-39, 1.7%; 40-64, 5.2%; 65+, 9.0%. The Laboratory-based Acute Physiology Score, version 2 (LAPS2) is assigned based on a patient's worst vital signs, pulse oximetry, neurological status, and 16 laboratory test results in the preceding 24 (discharge LAPS2) or 72 hours (admission LAPS2). The univariate relationship of an admission LAPS2 with 30-day mortality is as follows: 0-59, 1.0%; 60-109, 5.0%; 110+, 13.7%; for LAPS2dc, the relationship is 0-59, 2.2%; 60-109, 8.1%; 110+, 20.5%.

g Refers to disposition among patients discharged alive from the hospital. SNF = skilled nursing facility. Note that hospice referral is independent of discharge disposition.

h Transports-in (footnote c) are excluded from inpatient and 30-day mortality. Only patients who survived to discharge are included in the post-discharge outcomes. Non-elective rehospitalizations are those that began in the ED, were for an ambulatory care sensitive condition, and/or had a LAPS2 ≥ 60, as described in Escobar et al. (2015). Composite outcome = non-elective rehospitalization or death within 30 days after discharge.

©2019 Escobar GJ et al. *JAMA Network Open*

Table S1A-2: Patients with congestive heart failure (CHF)[a]					
TIME PERIOD	*6/10-5/11*	*6/11-9/12*	*10/12-12/14*	*1/15-12/17*	*TOTAL*
	Before HRRP penalty phase		***HRRP penalties in effect***		
N (hospitalizations)	6,018	7,501	14,077	17,511	45,107
N (patients)	4,839	5,719	10,154	12,735	28,912
Inpatient (INP) (%)[b]	92.4 (82.1 - 97.3)	85.7 (60.8 - 97.4)	83.3 (68.7 - 91.4)	79.2 (57.3 - 90.2)	83.3 (72.3 - 91.0)
Observation (OBS) (%)	7.6 (2.7 - 17.9)	14.3 (2.6 - 39.2)	16.7 (8.6 - 31.3)	20.8 (9.8 - 42.7)	16.7 (9.0 - 27.7)
Inpatient stay < 24 hours	4.3 (0.7 - 8.8)	3.9 (1.6 - 7.0)	3.0 (1.3 - 5.6)	1.8 (0.2 - 3.9)	2.9 (1.4 - 4.5)
Transport-in[c]	5.0 (0.5 - 10.9)	5.0 (0.6 - 14.0)	4.5 (1.3 - 9.2)	4.2 (0.3 - 8.6)	4.5 (1.2 - 9.6)
Age (mean)	75.3 (71.3 - 80.0)	75.0 (72.0 - 79.4)	75.1 (70.5 - 79.8)	74.3 (70.2 - 79.2)	74.8 (71.0 - 79.3)
Sex (% male)	50.7 (44.9 - 60.6)	51.8 (44.9 - 59.3)	52.2 (43.8 - 59.7)	53.3 (47.4 - 58.7)	52.4 (47.0 - 56.2)
Kaiser Foundation Health Plan, Inc. member (%)	94.5 (87.1 - 99.5)	94.7 (87.0 - 99.2)	93.9 (82.0 - 99.1)	91.0 (65.5 - 98.0)	93.0 (77.0 - 98.8)
Met strict HEDIS membership definition[d] (%)	78.4 (72.3 - 83.3)	79.5 (68.8 - 87.9)	77.7 (64.8 - 84.3)	74.7 (53.3 - 82.5)	76.9 (63.5 - 83.5)
Met public reporting definition[d] (%)	68.8 (57.5 - 78.2)	64.8 (45.6 - 77.1)	61.9 (42.3 - 73.8)	57.1 (40.5 - 69.1)	61.4 (46.4 - 72.6)
Hospitalization via emergency department (%)	91.3 (83.1 - 96.7)	92.7 (83.2 - 98.6)	94.3 (85.0 - 99.0)	94.9 (86.7 - 99.3)	93.9 (85.4 - 98.6)
Charlson score[e] (median)	4.0 (3.0 - 5.0)	5.0 (4.0 - 5.0)	5.0 (4.0 - 6.0)	5.0 (4.0 - 6.0)	5.0 (4.0 - 5.0)
Charlson score ≥ 4 (%)	61.4 (48.0 - 72.1)	65.4 (57.0 - 77.2)	66.5 (59.3 - 75.1)	67.8 (58.5 - 78.0)	66.1 (59.3 - 76.0)
COPS2[f] (mean)	67.8 (59.3 - 74.2)	74.3 (63.9 - 83.0)	80.1 (71.3 - 94.1)	79.3 (62.7 - 89.1)	77.2 (68.8 - 84.1)
COPS2 ≥ 65 (%)	49.0 (39.9 - 56.0)	55.0 (47.0 - 61.4)	59.1 (50.7 - 69.8)	58.2 (43.4 - 64.8)	56.7 (48.7 - 62.7)
Admission LAPS2[f] (mean)	83.9 (78.2 - 89.9)	84.8 (78.5 - 95.0)	85.7 (77.1 - 92.1)	84.8 (78.7 - 91.1)	84.9 (78.4 - 90.1)
Discharge LAPS2 (mean)	71.3 (60.1 - 79.3)	71.4 (64.3 - 76.3)	70.6 (62.0 - 75.3)	69.1 (61.4 - 75.1)	70.2 (62.0 - 74.1)
LAPS2 ≥ 110 (%)	20.0 (10.9 - 26.6)	19.9 (12.8 - 33.3)	20.8 (12.9 - 30.4)	19.8 (14.3 - 28.9)	20.2 (15.2 - 27.7)
Full code at discharge (%)	64.7 (39.8 - 73.6)	65.4 (48.9 - 78.9)	65.3 (50.0 - 77.1)	67.6 (49.1 - 84.0)	66.2 (50.9 - 79.7)
Length of stay (days) (mean)	5.0 (3.8 - 6.2)	5.2 (3.6 - 6.9)	4.8 (3.9 - 6.2)	5.0 (4.3 - 6.5)	5.0 (4.2 - 6.2)
Discharge disposition[g] (%)					
Regular Discharge (%)	74.0 (64.4 - 86.0)	72.9 (64.5 - 84.5)	71.0 (56.1 - 83.6)	70.1 (50.0 - 85.2)	71.4 (56.5 - 83.8)
Home Health (%)	14.6 (5.6 - 26.9)	17.1 (6.7 - 28.3)	19.4 (7.9 - 34.3)	21.3 (7.0 - 38.1)	19.1 (8.4 - 33.6)

©2019 Escobar GJ et al. *JAMA Network Open*

Table S1A-2: Patients with congestive heart failure (CHF)[a]					
TIME PERIOD	*6/10-5/11*	*6/11-9/12*	*10/12-12/14*	*1/15-12/17*	*TOTAL*
	Before HRRP penalty phase		***HRRP penalties in effect***		
Regular SNF (%)	9.2 (2.5 - 18.2)	8.5 (3.8 - 15.2)	8.0 (3.4 - 15.3)	7.4 (3.8 - 12.8)	8.0 (4.1 - 14.6)
Custodial SNF (%)	2.2 (0.4 - 5.0)	1.5 (0.3 - 5.4)	1.7 (0.3 - 2.9)	1.2 (0.1 - 3.6)	1.5 (0.4 - 2.8)
Hospice referral (%)	4.9 (2.8 - 9.0)	5.3 (2.6 - 11.1)	5.2 (2.7 - 10.2)	4.9 (1.5 - 12.5)	5.1 (2.3 - 9.7)
Outcomes[h]					
Inpatient mortality (%)	3.7 (1.2 - 6.3)	3.3 (0.7 - 7.5)	3.2 (1.6 - 5.2)	3.0 (1.7 - 4.5)	3.2 (1.7 - 4.5)
30-day mortality (%)	10.4 (6.2 - 14.7)	10.0 (3.5 - 17.4)	10.2 (6.5 - 13.6)	9.6 (6.2 - 12.9)	9.9 (7.0 - 12.5)
Any rehospitalization (%)	23.0 (15.0 - 28.4)	24.8 (18.4 - 30.5)	22.9 (16.3 - 27.7)	23.1 (17.4 - 29.7)	23.3 (18.2 - 28.6)
Any non-elective rehospitalization (%)	21.8 (13.9 - 26.4)	23.4 (16.7 - 29.4)	21.5 (15.5 - 26.2)	21.8 (15.7 - 27.7)	22.0 (16.3 - 26.9)
Non-elective inpatient rehospitalization (%)	20.2 (13.9 - 26.1)	20.7 (12.3 - 26.2)	18.7 (13.1 - 23.6)	17.8 (12.6 - 25.1)	18.9 (13.1 - 24.5)
Non-elective observation rehospitalization (%)	2.1 (0.7 - 4.8)	3.4 (0.5 - 7.1)	3.9 (1.4 - 8.0)	5.3 (1.7 - 9.9)	4.1 (2.2 - 6.9)
30-day post-discharge mortality (%)	8.3 (5.0 - 12.0)	8.2 (5.1 - 13.4)	8.3 (5.1 - 11.3)	7.8 (4.1 - 12.1)	8.1 (5.7 - 11.5)
Composite outcome (%)	27.0 (19.4 - 33.0)	28.4 (21.1 - 33.1)	27.0 (22.5 - 30.8)	27.0 (21.7 - 32.5)	27.2 (23.3 - 31.0)

F OOTNOTES

a Rates employ hospitalization episodes (which can include linked stays for patients who were transported) as the denominator. Numbers in parentheses always refer to the range across the 21 study hospitals. The period preceding the penalty phase of the Hospital Readmissions Reductions Program (HRRP), which took effect on 10/1/12, is divided into two epochs, with the first of these constituting the reference period for multivariate analyses and other comparisons. The period after the penalty phase also is divided into two epochs, with the first of these (through the end of 2014) matching that of the study of Gupta et al. and the second the remaining years.

b Hospital episodes where patients transitioned from OBS to INP status are classified as INP.

c Refers to patients whose linked hospitalization episode began at a hospital not owned by Kaiser Foundation Hospitals, Inc. These hospitalizations, involving 1,800 patients, had elevated inpatient (3.5%) and 30-day (11.1%) mortality, compared to 3.2% and 9.9% in the rest of the KPNC CHF cohort. Since we lacked information on their initial illness severity (the most important component of our mortality risk adjustment model) they are not included in inpatient and 30-day mortality analyses but are included in rehospitalization analyses, where admission illness severity plays a smaller role. See Escobar et al., 2008, 2013 and 2015, for details.

d The HEDIS membership definition restricts the denominator to patients with continuous health plan membership in the 12 months preceding and the 30 days following hospital discharge, with a maximum gap in coverage of 45 days in the preceding 12 months. The public reporting definition only includes patients meeting HEDIS membership criteria and excludes OBS hospitalizations and INP hospitalizations with length of stay < 24 hours.

e Charlson score was calculated using the methodology of Deyo et al. (1992).

f The COmorbidity Point Score, version 2 (COPS2) is assigned based on all diagnoses incurred by a patient in the 12 months preceding the index hospitalization. The univariate relationship of COPS2 with 30-day mortality is as follows: 0-39, 1.7%; 40-64, 5.2%; 65+, 9.0%. The Laboratory-based Acute Physiology Score, version 2 (LAPS2) is assigned based on a patient's worst vital signs, pulse oximetry, neurological status, and 16 laboratory test results in the preceding 24 (discharge LAPS2) or 72 hours (admission LAPS2). The univariate relationship of an admission LAPS2 with 30-day mortality is as follows: 0-59, 1.0%; 60-109, 5.0%; 110+, 13.7%; for LAPS2dc, the relationship is 0-59, 2.2%; 60-109, 8.1%; 110+, 20.5%.

g Refers to disposition among patients discharged alive from the hospital. SNF = skilled nursing facility. Note that hospice referral is independent of discharge disposition.

h Transports-in (footnote c) are excluded from inpatient and 30-day mortality. Only patients who survived to discharge are included in the post-discharge outcomes. Non-elective rehospitalizations are those that began in the ED, were for an ambulatory care sensitive condition, and/or had a LAPS2 ≥ 60, as described in Escobar et al. (2015). Composite outcome = non-elective rehospitalization or death within 30 days after discharge.

Table S1A-3: Patients with acute myocardial infarction (AMI)[a]					
TIME PERIOD	*6/10-5/11*	*6/11-9/12*	*10/12-12/14*	*1/15-12/17*	*TOTAL*
	Before HRRP penalty phase		***HRRP penalties in effect***		
N (hospitalizations)	4,194	5,471	10,131	15,684	35,480
N (patients)	3,945	5,158	9,348	14,339	31,187
Inpatient (INP) (%)[b]	96.2 (89.6 - 99.2)	94.8 (82.7 - 100)	90.5 (75.9 - 97.5)	84.1 (49.2 - 94.0)	89.0 (70.5 - 95.2)
Observation (OBS) (%)	3.8 (0.8 - 10.4)	5.2 (0.5 - 17.3)	9.5 (2.5 - 24.1)	15.9 (6.0 - 50.8)	11.0 (4.8 - 29.5)
Inpatient stay < 24 hours	7.0 (2.5 - 14.0)	8.0 (2.1 - 17.4)	7.0 (2.1 - 15.4)	5.3 (2.3 - 14.3)	6.4 (2.4 - 11.5)
Transport-in[c]	13.5 (1.3 - 28.2)	12.8 (3.1 - 33.3)	11.3 (2.3 - 29.2)	9.3 (0.3 - 20.6)	10.9 (3.6 - 26.1)
Age (mean)	69.6 (66.6 - 73.1)	69.0 (66.4 - 71.8)	69.3 (66.5 - 73.7)	68.4 (65.5 - 76.2)	68.9 (66.4 - 72.5)
Sex (% male)	62.0 (48.9 - 70.6)	62.5 (47.8 - 70.9)	63.1 (50.3 - 70.0)	63.6 (55.9 - 71.9)	63.1 (54.7 - 69.7)
Kaiser Foundation Health Plan, Inc. member (%)	94.5 (86.4 - 98.8)	94.4 (86.0 - 100)	93.0 (80.6 - 98.7)	90.5 (68.9 - 98.1)	92.3 (80.7 - 97.6)
Met strict HEDIS membership definition[d] (%)	81.7 (74.0 - 87.4)	82.3 (72.8 - 87.6)	80.3 (69.0 - 88.0)	77.8 (59.6 - 84.8)	79.6 (70.0 - 85.9)
Met public reporting definition[d] (%)	73.4 (65.0 - 81.7)	72.2 (58.0 - 78.8)	67.5 (52.3 - 78.1)	61.2 (36.7 - 73.5)	66.1 (54.5 - 76.9)
Hospitalization via emergency department (%)	82.0 (39.4 - 98.0)	82.9 (50.1 - 95.9)	84.4 (52.4 - 97.7)	87.5 (62.6 - 99.5)	85.3 (53.9 - 96.6)
Charlson score[e] (median)	2.0 (1.0 - 3.0)	2.0 (1.0 – 4.0)	2.0 (1.0 – 4.0)	2.0 (1.0 - 4.5)	2.0 (1.0 - 4.0)
Charlson score ≥ 4 (%)	32.8 (20.4 - 47.4)	32.9 (22.2 - 57.8)	35.8 (28.1 - 52.3)	36.9 (26.2 - 61.8)	35.5 (28.1 - 51.5)
COPS2[f] (mean)	32.2 (25.3 - 42.1)	33.3 (27.3 - 49.8)	37.9 (30.4 - 48.6)	37.8 (28.8 - 59.9)	36.5 (31.2 - 47.3)
COPS2 ≥ 65 (%)	16.7 (9.2 - 31.7)	16.7 (11.4 - 31.1)	20.6 (15.1 - 36.4)	20.3 (11.7 - 50.0)	19.4 (15.2 - 33.9)
Admission LAPS2[f] (mean)	70.5 (60.1 - 84.9)	70.2 (59.2 - 82.8)	71.1 (62.4 - 88.0)	69.3 (58.7 - 103)	70.1 (61.7 - 87.1)
Discharge LAPS2 (mean)	56.6 (47.1 - 62.9)	55.4 (44.4 - 70.0)	53.2 (46.8 - 62.0)	52.5 (45.6 - 65.6)	53.7 (49.8 - 62.8)
LAPS2 ≥ 110 (%)	14.5 (5.4 - 24.0)	13.5 (6.3 - 25.4)	14.3 (9.3 - 22.1)	12.6 (8.0 - 37.5)	13.5 (9.5 - 21.1)
Full code at discharge (%)	85.9 (79.1 - 92.9)	87.0 (78.3 - 93.8)	86.8 (69.2 - 92.9)	89.0 (67.7 - 92.3)	87.7 (78.1 - 92.0)
Length of stay (days) (mean)	5.2 (4.3 - 6.7)	4.9 (4.2 - 5.7)	4.7 (4.3 - 5.8)	4.7 (4.0 - 5.5)	4.8 (4.3 - 5.4)
Discharge disposition[g] (%)					
Regular Discharge (%)	82.8 (68.8 - 89.1)	83.9 (78.0 - 87.2)	83.2 (74.4 - 89.5)	84.5 (71.0 - 91.0)	83.8 (76.9 - 88.9)
Home Health (%)	9.5 (5.8 - 16.3)	10.4 (5.8 - 15.8)	11.6 (6.7 - 15.9)	11.2 (5.7 - 22.6)	11.0 (6.8 - 16.2)

©2019 Escobar GJ et al. *JAMA Network Open*

Table S1A-3: Patients with acute myocardial infarction (AMI)[a]					
TIME PERIOD	*6/10-5/11*	*6/11-9/12*	*10/12-12/14*	*1/15-12/17*	*TOTAL*
	Before HRRP penalty phase		***HRRP penalties in effect***		
Regular SNF (%)	6.7 (2.4 - 14.0)	4.9 (2.6 - 8.8)	4.5 (1.6 - 8.5)	3.7 (1.6 - 6.5)	4.5 (2.4 - 7.3)
Custodial SNF (%)	1.0 (0.3 - 5.7)	0.8 (0.3 - 4.5)	0.8 (0.3 - 2.6)	0.5 (0.1 - 1.4)	0.7 (0.3 - 2.5)
Hospice referral (%)	1.8 (0.3 - 6.6)	1.8 (0.3 - 4.5)	2.6 (0.6 - 13.6)	2.0 (0.7 - 8.8)	2.1 (0.8 - 6.7)
Outcomes[h]					
Inpatient mortality (%)	4.8 (2.1 - 9.3)	4.5 (0.6 - 8.6)	4.3 (2.7 - 11.6)	4.1 (1.5 - 6.8)	4.3 (2.0 - 6.9)
30-day mortality (%)	7.6 (2.8 - 13.3)	7.5 (2.7 - 15.3)	7.4 (4.5 - 20.9)	6.7 (4.0 - 25.0)	7.1 (4.6 - 12.9)
Any rehospitalization (%)	16.7 (10.9 - 22.3)	15.2 (10.6 - 26.4)	14.0 (9.6 - 23.1)	13.6 (10.2 - 29.0)	14.3 (11.3 - 18.6)
Any non-elective rehospitalization (%)	14.8 (10.3 - 20.4)	13.6 (7.7 - 24.0)	12.5 (9.0 - 23.1)	12.1 (7.3 - 29.0)	12.8 (8.7 - 17.5)
Non-elective inpatient rehospitalization (%)	13.2 (8.7 - 19.1)	10.9 (6.7 - 16.8)	10.1 (7.3 - 23.1)	8.9 (5.9 - 22.6)	10.0 (7.6 - 15.0)
Non-elective observation rehospitalization (%)	2.1 (0.5 - 4.3)	3.4 (0.7 - 9.4)	2.9 (1.5 - 5.2)	4.1 (1.0 - 9.7)	3.4 (1.5 - 6.0)
30-day post-discharge mortality (%)	3.8 (0.3 - 8.6)	3.5 (1.2 - 6.5)	3.6 (1.7 - 12.8)	3.1 (1.6 – 22.6)	3.4 (1.7 – 8.1)
Composite outcome (%)	17.4 (10.9 - 27.4)	16.2 (12.1 - 26.4)	14.9 (11.5 - 35.9)	14.2 (11.0 – 45.2)	15.1 (12.5 – 24.4)

FOOTNOTES

a Rates employ hospitalization episodes (which can include linked stays for patients who were transported) as the denominator. Numbers in parentheses always refer to the range across the 21 study hospitals. The period preceding the penalty phase of the Hospital Readmissions Reductions Program (HRRP), which took effect on 10/1/12, is divided into two epochs, with the first of these constituting the reference period for multivariate analyses and other comparisons. The period after the penalty phase also is divided into two epochs, with the first of these (through the end of 2014) matching that of the study of Gupta et al. and the second the remaining years.

b Hospital episodes where patients transitioned from OBS to INP status are classified as INP.

c Refers to patients whose linked hospitalization episode began at a hospital not owned by Kaiser Foundation Hospitals, Inc. These hospitalizations, involving 3,762 patients, had lower inpatient (3.8%) and 30-day (6.8%) mortality, compared to 4.3% and 7.1% in the rest of the KPNC AMI cohort. Since we lacked information on their initial illness severity (the most important component of our mortality risk adjustment model) they are not included in inpatient and 30-day mortality analyses but are included in rehospitalization analyses, where admission illness severity plays a smaller role. See Escobar et al., 2008, 2013 and 2015, for details.

d The HEDIS membership definition restricts the denominator to patients with continuous health plan membership in the 12 months preceding and the 30 days following hospital discharge, with a maximum gap in coverage of 45 days in the preceding 12 months. The public reporting definition only includes patients meeting HEDIS membership criteria and excludes OBS hospitalizations and INP hospitalizations with length of stay < 24 hours.

e Charlson score was calculated using the methodology of Deyo et al. (1992).

©2019 Escobar GJ et al. *JAMA Network Open*

f The COmorbidity Point Score, version 2 (COPS2) is assigned based on all diagnoses incurred by a patient in the 12 months preceding the index hospitalization. The univariate relationship of COPS2 with 30-day mortality is as follows: 0-39, 1.7%; 40-64, 5.2%; 65+, 9.0%. The Laboratory-based Acute Physiology Score, version 2 (LAPS2) is assigned based on a patient's worst vital signs, pulse oximetry, neurological status, and 16 laboratory test results in the preceding 24 (discharge LAPS2) or 72 hours (admission LAPS2). The univariate relationship of an admission LAPS2 with 30-day mortality is as follows: 0-59, 1.0%; 60-109, 5.0%; 110+, 13.7%; for LAPS2dc, the relationship is 0-59, 2.2%; 60-109, 8.1%; 110+, 20.5%.

g Refers to disposition among patients discharged alive from the hospital. SNF = skilled nursing facility. Note that hospice referral is independent of discharge disposition.

h Transports-in (footnote c) are excluded from inpatient and 30-day mortality. Only patients who survived to discharge are included in the post-discharge outcomes. Non-elective rehospitalizations are those that began in the ED, were for an ambulatory care sensitive condition, and/or had a LAPS2 ≥ 60, as described in Escobar et al. (2015). Composite outcome = non-elective rehospitalization or death within 30 days after discharge.

©2019 Escobar GJ et al. *JAMA Network Open*

Table S1A-4: Patients without CAP, CHF, or AMI[a]					
TIME PERIOD	*6/10-5/11*	*6/11-9/12*	*10/12-12/14*	*1/15-12/17*	*TOTAL*
	Before HRRP penalty phase		***HRRP penalties in effect***		
N (hospitalizations)	160,693	213,952	360,529	544,319	1,279,493
N (patients)	120,235	153,741	238,734	335,698	656,338
Inpatient (INP) (%)[b]	90.3 (81.9 - 95.4)	87.0 (78.7 - 94.0)	83.9 (71.7 - 91.4)	79.5 (58.5 - 89.6)	83.4 (71.1 - 90.7)
Observation (OBS) (%)	9.7 (4.6 - 18.1)	13.0 (6.0 - 21.3)	16.1 (8.6 - 28.3)	20.5 (10.4 - 41.5)	16.6 (9.3 - 28.9)
Inpatient stay < 24 hours	7.0 (4.5 - 9.4)	6.0 (4.1 - 7.9)	4.8 (3.1 - 6.5)	4.7 (2.0 - 7.0)	5.2 (3.6 - 6.9)
Transport-in[c]	4.4 (1.2 - 7.8)	4.4 (1.5 - 8.3)	4.1 (1.9 - 8.5)	4.3 (0.3 - 8.6)	4.3 (1.4 - 8.4)
Age (mean)	63.8 (59.6 - 68.8)	63.9 (59.8 - 68.4)	64.6 (61.2 - 69.2)	65.1 (62.2 - 69.8)	64.6 (61.4 - 69.2)
Sex (% male)	45.3 (40.0 - 52.3)	45.7 (40.0 - 52.5)	46.5 (41.4 - 53.1)	47.6 (43.6 - 53.1)	46.7 (42.0 - 52.9)
Kaiser Foundation Health Plan, Inc. member (%)	94.7 (86.1 - 97.7)	94.6 (84.2 - 97.9)	94.2 (84.5 - 98.1)	92.8 (64.9 - 97.7)	93.8 (77.3 - 97.9)
Met strict HEDIS membership definition[d] (%)	82.0 (74.1 - 85.3)	82.3 (72.4 - 86.7)	81.1 (71.6 - 85.8)	79.0 (54.0 - 84.9)	80.5 (65.1 - 85.2)
Met public reporting definition[d] (%)	68.4 (59.2 - 75.9)	66.9 (55.4 - 76.2)	64.2 (52.1 - 72.3)	58.7 (39.5 - 69.5)	62.8 (48.0 - 70.3)
Hospitalization via emergency department (%)	64.7 (50.5 - 84.5)	65.5 (54.3 - 79.0)	67.3 (55.0 - 80.4)	70.0 (54.2 - 85.2)	67.8 (54.4 - 79.9)
Charlson score[e] (median)	2.0 (1.0 - 2.0)	2.0 (1.0 - 2.0)	2.0 (1.0 – 2.0)	2.0 (2.0 - 3.0)	2.0 (1.0 - 2.0)
Charlson score ≥ 4 (%)	27.2 (23.6 - 32.7)	28.9 (24.1 - 35.3)	31.7 (26.1 - 38.9)	37.2 (31.1 - 42.7)	33.0 (27.6 - 38.6)
COPS2[f] (mean)	35.9 (33.3 - 43.0)	38.6 (34.6 - 46.0)	43.9 (38.1 - 52.8)	46.8 (38.6 - 52.3)	43.2 (37.4 - 50.3)
COPS2 ≥ 65 (%)	19.3 (17.4 - 25.0)	21.2 (18.0 - 27.0)	25.3 (20.6 - 32.2)	27.8 (21.4 - 32.1)	24.9 (20.1 - 30.3)
Admission LAPS2[f] (mean)	53.1 (44.0 - 62.2)	53.9 (44.5 - 62.5)	55.9 (45.1 - 65.6)	58.3 (47.1 - 68.9)	56.3 (45.7 - 65.5)
Discharge LAPS2 (mean)	44.8 (39.1 - 50.1)	45.1 (41.7 - 50.1)	44.8 (41.5 - 49.8)	45.8 (41.0 - 50.3)	45.3 (41.5 - 49.5)
LAPS2 ≥ 110 (%)	9.6 (6.7 - 14.4)	10.3 (6.7 - 14.4)	11.2 (6.6 - 15.4)	12.2 (7.8 - 16.5)	11.3 (7.1 - 15.4)
Full code at discharge (%)	86.2 (78.6 - 91.1)	85.8 (78.7 - 92.2)	85.1 (78.7 - 91.5)	85.0 (78.0 - 90.8)	85.3 (78.4 - 91.2)
Length of stay (days) (mean)	5.0 (4.2 - 5.7)	5.0 (4.0 – 5.5)	4.9 (3.7 - 5.5)	4.8 (3.8 - 5.5)	4.9 (3.9 - 5.4)
Discharge disposition[g] (%)					
Regular Discharge (%)	75.8 (71.9 - 85.5)	74.3 (68.9 - 85.2)	72.3 (65.5 - 85.0)	71.5 (52.7 - 87.7)	72.7 (62.0 - 86.3)
Home Health (%)	12.3 (6.6 - 17.9)	13.3 (7.0 - 18.9)	15.9 (7.1 - 23.4)	17.9 (6.1 - 31.4)	15.8 (6.6 - 22.8)

©2019 Escobar GJ et al. *JAMA Network Open*

Table S1A-4: Patients without CAP, CHF, or AMI[a]					
TIME PERIOD	*6/10-5/11*	*6/11-9/12*	*10/12-12/14*	*1/15-12/17*	*TOTAL*
	Before HRRP penalty phase		***HRRP penalties in effect***		
Regular SNF (%)	10.3 (6.9 - 15.8)	10.9 (6.4 - 15.6)	10.3 (6.6 - 16.0)	9.5 (5.4 - 13.7)	10.1 (6.0 - 14.9)
Custodial SNF (%)	1.6 (0.8 - 2.4)	1.6 (0.9 - 3.1)	1.5 (0.7 - 2.8)	1.1 (0.5 - 2.2)	1.4 (0.8 - 2.5)
Hospice referral (%)	2.4 (1.7 - 3.7)	2.4 (1.5 - 4.2)	2.5 (1.7 - 4.7)	2.5 (1.6 - 4.0)	2.5 (1.6 - 4.2)
Outcomes[h]					
Inpatient mortality (%)	2.7 (1.9 - 3.6)	2.6 (1.9 – 3.5)	2.7 (1.9 - 3.4)	2.7 (2.0 - 3.3)	2.7 (2.0 - 3.2)
30-day mortality (%)	5.6 (3.6 – 7.0)	5.7 (3.8 - 7.1)	5.8 (4.0 - 7.5)	6.0 (4.0 – 7.4)	5.8 (3.9 – 7.3)
Any rehospitalization (%)	14.0 (12.0 - 16.6)	13.9 (11.8 - 16.8)	13.7 (10.7 - 16.9)	14.3 (12.2 - 16.6)	14.0 (11.8 - 16.7)
Any non-elective rehospitalization (%)	11.5 (9.2 - 14.6)	11.5 (9.4 - 14.7)	11.5 (9.5 - 14.9)	12.2 (10.1 - 14.9)	11.8 (10.0 - 14.9)
Non-elective inpatient rehospitalization (%)	10.6 (8.6 - 13.8)	10.2 (7.6 - 13.2)	9.9 (7.4 - 12.3)	10.1 (8.1 - 12.2)	10.1 (7.9 - 12.3)
Non-elective observation rehospitalization (%)	1.2 (0.7 - 1.7)	1.8 (0.6 - 3.0)	2.2 (1.0 - 3.3)	2.8 (1.7 - 5.0)	2.2 (1.2 - 3.3)
30-day post-discharge mortality (%)	3.7 (2.3 - 5.1)	3.8 (2.7 - 5.0)	3.9 (2.7 - 5.3)	4.0 (2.5 - 5.1)	3.9 (2.6 - 5.2)
Composite outcome (%)	14.1 (10.9 - 18.3)	14.2 (12.1 - 18.2)	14.3 (12.2 - 18.5)	15.0 (12.7 - 18.2)	14.6 (12.4 - 18.3)

FOOTNOTES

a Rates employ hospitalization episodes (which can include linked stays for patients who were transported) as the denominator. Numbers in parentheses always refer to the range across the 21 study hospitals. The period preceding the penalty phase of the Hospital Readmissions Reductions Program (HRRP), which took effect on 10/1/12, is divided into two epochs, with the first of these constituting the reference period for multivariate analyses and other comparisons. The period after the penalty phase also is divided into two epochs, with the first of these (through the end of 2014) matching that of the study of Gupta et al. and the second the remaining years.

b Hospital episodes where patients transitioned from OBS to INP status are classified as INP.

c Refers to patients whose linked hospitalization episode began at a hospital not owned by Kaiser Foundation Hospitals, Inc. These hospitalizations, involving 47,514 patients, had elevated inpatient (5.1%) and 30-day (9.5%) mortality, compared to 2.7% and 5.8% in the rest of the KPNC NCCA cohort. Since we lacked information on their initial illness severity (the most important component of our mortality risk adjustment model) they are not included in inpatient and 30-day mortality analyses but are included in rehospitalization analyses, where admission illness severity plays a smaller role. See Escobar et al., 2008, 2013 and 2015, for details.

d The HEDIS membership definition restricts the denominator to patients with continuous health plan membership in the 12 months preceding and the 30 days following hospital discharge, with a maximum gap in coverage of 45 days in the preceding 12 months. The public reporting definition only includes patients meeting HEDIS membership criteria and excludes OBS hospitalizations and INP hospitalizations with length of stay < 24 hours.

e Charlson score was calculated using the methodology of Deyo et al. (1992).

©2019 Escobar GJ et al. *JAMA Network Open*

f The COmorbidity Point Score, version 2 (COPS2) is assigned based on all diagnoses incurred by a patient in the 12 months preceding the index hospitalization. The univariate relationship of COPS2 with 30-day mortality is as follows: 0-39, 1.7%; 40-64, 5.2%; 65+, 9.0%. The Laboratory-based Acute Physiology Score, version 2 (LAPS2) is assigned based on a patient's worst vital signs, pulse oximetry, neurological status, and 16 laboratory test results in the preceding 24 (discharge LAPS2) or 72 hours (admission LAPS2). The univariate relationship of an admission LAPS2 with 30-day mortality is as follows: 0-59, 1.0%; 60-109, 5.0%; 110+, 13.7%; for LAPS2dc, the relationship is 0-59, 2.2%; 60-109, 8.1%; 110+, 20.5%.

g Refers to disposition among patients discharged alive from the hospital. SNF = skilled nursing facility. Note that hospice referral is independent of discharge disposition.

h Transports-in (footnote c) are excluded from inpatient and 30-day mortality. Only patients who survived to discharge are included in the post-discharge outcomes. Non-elective rehospitalizations are those that began in the ED, were for an ambulatory care sensitive condition, and/or had a LAPS2 ≥ 60, as described in Escobar et al. (2015). Composite outcome = non-elective rehospitalization or death within 30 days after discharge.

©2019 Escobar GJ et al. *JAMA Network Open*

Table S1A-5: Inpatients only[a]					
TIME PERIOD	*6/10-5/11*	*6/11-9/12*	*10/12-12/14*	*1/15-12/17*	*TOTAL*
	Before HRRP penalty phase		***HRRP penalties in effect***		
N (hospitalizations)	158,787	201,104	327,950	467,193	1,155,034
N (patients)	117,426	144,877	219,605	297,082	607,801
Inpatient stay < 24 hours	7.6 (4.6 - 10.3)	6.8 (4.7 - 9.0)	5.7 (4.1 - 7.6)	5.8 (3.0 - 8.5)	6.2 (4.2 - 8.1)
Transport-in[b]	4.7 (1.2 - 8.6)	4.7 (1.6 - 8.9)	4.4 (2.2 - 9.0)	4.6 (0.4 - 9.0)	4.6 (1.6 - 8.8)
Age (mean)	64.4 (60.4 – 68.8)	64.3 (60.0 - 68.5)	65.0 (61.6 - 68.9)	65.4 (62.2 - 69.5)	65.0 (61.7 - 69.1)
Sex (% male)	46.1 (40.7 - 53.1)	46.6 (39.9 - 53.3)	47.4 (41.1 - 54.2)	48.3 (43.2 - 54.8)	47.4 (41.8 - 54.1)
Kaiser Foundation Health Plan, Inc. member (%)	94.8 (86.3 - 97.8)	94.8 (84.9 - 98.0)	94.3 (84.7 - 98.2)	92.7 (65.4 - 97.7)	93.8 (77.7 - 97.9)
Met strict HEDIS membership definition[c] (%)	81.5 (73.8 - 85.2)	82.0 (72.4 - 86.4)	80.6 (70.8 - 85.2)	78.2 (53.8 - 84.0)	80.0 (64.7 - 84.8)
Met public reporting definition[c] (%)	75.7 (66.9 - 80.8)	76.8 (67.1 - 82.5)	76.4 (66.1 - 81.5)	73.8 (49.7 - 80.5)	75.3 (59.8 - 81.2)
Hospitalization via emergency department (%)	64.4 (51.3 - 84.5)	64.0 (52.1 - 78.2)	65.7 (53.1 - 78.3)	67.6 (52.6 - 84.5)	66.0 (52.8 - 78.4)
Charlson score[d] (median)	2.0 (1.0 - 2.0)	2.0 (1.0 - 2.0)	2.0 (1.0 - 3.0)	2.0 (2.0 - 3.0)	2.0 (2.0 - 2.0)
Charlson score ≥ 4 (%)	28.9 (24.9 - 35.2)	30.1 (24.9 - 36.6)	33.2 (27.3 - 41.9)	38.2 (31.6 - 44.2)	34.1 (28.5 - 40.0)
COPS2[e] (mean)	37.5 (34.6 - 45.2)	40.0 (34.8 - 48.1)	45.5 (38.3 - 56.3)	48.1 (39.9 - 54.2)	44.5 (38.5 - 52.2)
COPS2 ≥ 65 (%)	20.8 (18.3 - 27.1)	22.5 (18.1 - 29.0)	26.7 (21.1 - 35.5)	29.0 (22.0 - 34.0)	26.1 (21.1 - 32.0)
Admission LAPS2[e] (mean)	55.5 (46.7 - 66.2)	55.7 (46.5 - 65.0)	58.0 (47.1 - 69.3)	60.4 (48.9 - 72.4)	58.2 (47.6 - 68.5)
Discharge LAPS2 (mean)	46.0 (41.1 - 51.9)	46.1 (42.9 - 50.8)	45.8 (41.9 - 51.1)	46.3 (41.2 - 51.1)	46.1 (41.9 - 50.0)
LAPS2 ≥ 110 (%)	11.0 (7.7 - 15.9)	11.6 (7.5 - 16.8)	12.8 (7.5 - 18.5)	14.0 (9.1 - 18.9)	12.8 (8.1 - 17.6)
Full code at discharge (%)	85.2 (78.9 - 89.6)	85.3 (78.6 - 92.4)	84.7 (79.5 - 90.4)	84.7 (78.4 - 90.3)	84.9 (78.8 - 90.6)
Length of stay (days) (mean)	5.3 (4.5 - 6.0)	5.3 (4.4 - 5.9)	5.3 (4.1 - 5.9)	5.3 (4.2 - 6.0)	5.3 (4.3 - 5.8)
Discharge disposition[f] (%)					
Regular Discharge (%)	74.4 (70.5 - 83.8)	72.6 (66.7 - 84.4)	70.1 (61.3 - 83.3)	68.5 (45.3 - 86.3)	70.5 (57.9 - 84.9)
Home Health (%)	13.1 (7.7 - 19.1)	14.3 (7.7 - 20.1)	17.3 (8.0 - 25.2)	19.8 (6.8 - 36.7)	17.2 (7.4 - 24.9)
Regular SNF (%)	10.8 (7.5 - 16.6)	11.4 (6.5 - 16.5)	11.0 (7.3 - 17.9)	10.4 (6.0 - 15.5)	10.8 (6.6 - 16.4)
Custodial SNF (%)	1.7 (1.0 - 2.7)	1.6 (0.9 - 3.5)	1.6 (0.8 - 3.1)	1.2 (0.5 - 2.6)	1.5 (0.8 - 2.9)

©2019 Escobar GJ et al. *JAMA Network Open*

Table S1A-5: Inpatients only[a]					
TIME PERIOD	*6/10-5/11*	*6/11-9/12*	*10/12-12/14*	*1/15-12/17*	*TOTAL*
	Before HRRP penalty phase		***HRRP penalties in effect***		
Hospice referral (%)	2.6 (1.8 - 4.0)	2.6 (1.7 - 4.6)	2.7 (1.8 - 5.4)	2.8 (1.7 - 4.7)	2.7 (1.8 - 4.8)
Outcomes[g]					
Inpatient mortality (%)	3.0 (2.0 - 4.4)	3.0 (2.1 – 4.2)	3.1 (2.1 - 3.8)	3.3 (2.4 - 4.0)	3.1 (2.3 – 3.7)
30-day mortality (%)	6.2 (3.9 - 8.0)	6.3 (4.1 - 8.0)	6.5 (4.3 – 8.5)	6.7 (4.5 - 8.7)	6.5 (4.3 – 8.2)
Any rehospitalization (%)	14.7 (12.7 - 17.1)	14.5 (11.9 - 17.9)	14.4 (11.6 - 18.5)	15.1 (12.7 - 18.1)	14.8 (12.6 - 17.9)
Any non-elective rehospitalization (%)	12.2 (10.1 - 15.1)	12.2 (9.6 - 15.8)	12.2 (10.0 - 16.4)	12.9 (10.4 - 16.6)	12.5 (10.6 - 15.9)
Non-elective inpatient rehospitalization (%)	11.3 (9.3 - 14.2)	10.9 (8.1 - 14.4)	10.6 (8.2 - 13.9)	10.9 (8.8 - 13.4)	10.9 (8.6 - 13.5)
Non-elective observation rehospitalization (%)	1.2 (0.7 - 1.7)	1.7 (0.5 - 2.8)	2.1 (1.0 - 3.3)	2.7 (1.6 - 5.0)	2.1 (1.2 - 3.2)
30-day post-discharge mortality (%)	4.1 (2.5 - 5.7)	4.2 (3.0 - 5.8)	4.3 (2.9 - 6.0)	4.4 (2.8 - 5.8)	4.3 (2.8 - 5.8)
Composite outcome (%)	15.1 (11.9 - 19.2)	15.1 (13.0 - 19.8)	15.3 (12.9 - 20.4)	16.0 (13.4 - 20.3)	15.5 (13.2 - 19.8)

FOOTNOTES

a Rates employ hospitalization episodes (which can include linked stays for patients who were transported) as the denominator. Numbers in parentheses always refer to the range across the 21 study hospitals. The period preceding the penalty phase of the Hospital Readmissions Reductions Program (HRRP), which took effect on 10/1/12, is divided into two epochs, with the first of these constituting the reference period for multivariate analyses and other comparisons. The period after the penalty phase also is divided into two epochs, with the first of these (through the end of 2014) matching that of the study of Gupta et al. and the second the remaining years.

b Refers to patients whose linked hospitalization episode began at a hospital not owned by Kaiser Foundation Hospitals, Inc. These hospitalizations, involving 46,072 patients, had elevated inpatient (5.6%) and 30-day (10.3%) mortality, compared to 3.1% and 6.5% in the rest of the KPNC INP cohort. Since we lacked information on their initial illness severity (the most important component of our mortality risk adjustment model) they are not included in inpatient and 30-day mortality analyses but are included in rehospitalization analyses, where admission illness severity plays a smaller role. See Escobar et al., 2008, 2013 and 2015, for details.

c The HEDIS membership definition restricts the denominator to patients with continuous health plan membership in the 12 months preceding and the 30 days following hospital discharge, with a maximum gap in coverage of 45 days in the preceding 12 months. The public reporting definition only includes patients meeting HEDIS membership criteria and excludes OBS hospitalizations and INP hospitalizations with length of stay < 24 hours.

d Charlson score was calculated using the methodology of Deyo et al. (1992).

e The COmorbidity Point Score, version 2 (COPS2) is assigned based on all diagnoses incurred by a patient in the 12 months preceding the index hospitalization. The univariate relationship of COPS2 with 30-day mortality is as follows: 0-39, 1.7%; 40-64, 5.2%; 65+, 9.0%. The Laboratory-based Acute Physiology Score, version 2 (LAPS2) is assigned based on a patient's worst vital signs, pulse oximetry, neurological status, and 16 laboratory test results in the preceding 24 (discharge LAPS2) or 72 hours (admission LAPS2). The univariate relationship of an admission LAPS2 with 30-day mortality is as follows: 0-59, 1.0%; 60-109, 5.0%; 110+, 13.7%; for LAPS2dc, the relationship is 0-59, 2.2%; 60-109, 8.1%; 110+, 20.5%.

©2019 Escobar GJ et al. *JAMA Network Open*

f Refers to disposition among patients discharged alive from the hospital. SNF = skilled nursing facility. Note that hospice referral is independent of discharge disposition.

g Transports-in (footnote b) are excluded from inpatient and 30-day mortality. Only patients who survived to discharge are included in the post-discharge outcomes. Non-elective rehospitalizations are those that began in the ED, were for an ambulatory care sensitive condition, and/or had a LAPS2 ≥ 60, as described in Escobar et al. (2015). Composite outcome = non-elective rehospitalization or death within 30 days after discharge.

©2019 Escobar GJ et al. *JAMA Network Open*

Table S1A-6: Observation hospitalizations only[a]					
TIME PERIOD	*6/10-5/11*	*6/11-9/12*	*10/12-12/14*	*1/15-12/17*	*TOTAL*
	Before HRRP penalty phase		***HRRP penalties in effect***		
N (hospitalizations)	16,497	29,679	62,600	120,215	228,991
N (patients)	15,633	26,906	54,450	98,849	177,129
Transport-in[b]	4.3 (0.3 - 8.7)	4.4 (1.2 - 8.5)	3.9 (1.6 - 8.7)	3.7 (0.2 - 7.3)	3.9 (1.2 - 7.8)
Age (mean)	66.0 (60.6 - 73.1)	66.3 (62.2 - 70.9)	66.5 (59.3 - 71.8)	66.3 (61.1 - 72.0)	66.3 (60.8 - 71.8)
Sex (% male)	44.5 (39.9 - 54.9)	44.9 (40.4 - 52.6)	45.7 (41.6 - 51.5)	47.7 (43.8 - 51.5)	46.6 (43.7 - 50.6)
Kaiser Foundation Health Plan, Inc. member (%)	93.9 (86.0 - 98.4)	93.4 (82.8 - 97.9)	93.8 (84.2 - 97.7)	92.8 (63.3 - 97.8)	93.2 (76.2 - 97.8)
Met strict HEDIS membership definition[c] (%)	84.3 (76.1 - 88.7)	83.5 (72.7 - 90.0)	82.6 (73.8 - 88.4)	81.3 (55.7 - 87.8)	82.2 (67.7 - 88.2)
Met public reporting definition[c] (%)	0	0	0	0	0
Hospitalization via emergency department (%)	88.8 (66.3 - 96.3)	89.3 (79.1 - 96.9)	87.4 (72.8 - 92.8)	87.0 (73.3 - 94.3)	87.5 (74.4 - 94.0)
Charlson score[d] (median)	2.0 (1.0 - 2.0)	2.0 (1.0 – 3.0)	2.0 (2.0 - 2.0)	2.0 (2.0 - 3.0)	2.0 (2.0 - 3.0)
Charlson score ≥ 4 (%)	28.1 (22.6 - 33.7)	31.9 (25.6 - 44.1)	33.7 (28.2 - 38.3)	38.9 (33.7 - 45.6)	35.8 (30.1 - 42.3)
COPS2[e] (mean)	34.9 (31.3 - 39.1)	39.6 (33.7 - 47.1)	44.7 (40.0 - 48.7)	46.9 (37.3 - 52.7)	44.5 (39.7 - 49.5)
COPS2 ≥ 65 (%)	17.9 (14.4 - 21.2)	21.9 (14.8 - 29.5)	26.2 (21.4 - 31.4)	28.1 (20.2 - 34.8)	26.1 (22.3 - 31.0)
Admission LAPS2[e] (mean)	53.1 (40.9 - 62.9)	55.7 (44.3 - 67.0)	56.6 (45.9 - 62.3)	57.4 (48.1 - 65.7)	56.7 (47.3 - 63.1)
Discharge LAPS2 (mean)	48.8 (41.1 - 57.0)	48.4 (42.7 - 53.1)	48.1 (43.3 - 53.5)	49.0 (42.1 - 55.6)	48.6 (43.9 - 54.3)
LAPS2 ≥ 110 (%)	4.1 (1.5 – 7.7)	5.5 (2.3 – 9.3)	6.3 (3.6 - 8.8)	6.8 (3.8 - 9.8)	6.3 (3.7 – 8.5)
Full code at discharge (%)	83.8 (69.8 - 91.4)	82.2 (74.2 - 88.2)	81.8 (72.7 - 91.4)	83.1 (73.3 - 90.5)	82.7 (73.0 - 90.4)
Length of stay (days) (mean)	2.4 (2.2 - 2.5)	2.6 (2.3 - 2.9)	2.7 (2.4 - 3.0)	2.7 (2.3 - 3.4)	2.7 (2.4 - 3.1)
Discharge disposition[f] (%)					
Regular Discharge (%)	89.5 (83.6 - 96.3)	86.3 (75.3 - 93.0)	84.6 (77.9 - 90.1)	84.1 (73.3 - 92.6)	84.9 (76.5 - 91.2)
Home Health (%)	5.2 (1.0 - 10.2)	6.9 (2.8 - 12.7)	8.7 (4.8 - 15.1)	10.1 (4.5 - 16.5)	9.0 (4.6 - 14.4)
Regular SNF (%)	4.4 (0.7 - 10.0)	5.7 (2.3 - 10.5)	5.5 (2.7 - 10.0)	4.9 (2.3 - 8.7)	5.1 (2.9 - 9.1)
Custodial SNF (%)	1.0 (0.2 - 2.3)	1.1 (0.5 - 2.0)	1.1 (0.1 - 2.3)	0.8 (0.2 - 1.8)	1.0 (0.5 - 2.0)
Hospice referral (%)	1.5 (0.4 - 3.3)	1.8 (1.1 - 3.5)	2.1 (1.0 - 3.4)	2.0 (0.7 - 3.5)	2.0 (1.0 - 3.1)

Table S1A-6: Observation hospitalizations only[a]					
TIME PERIOD	*6/10-5/11*	*6/11-9/12*	*10/12-12/14*	*1/15-12/17*	*TOTAL*
	Before HRRP penalty phase		***HRRP penalties in effect***		
Outcomes[g]					
Inpatient mortality (%)	0.6 (0.1 – 1.2)	0.8 (0.1 – 1.6)	1.1 (0.2 - 2.2)	1.0 (0.3 - 2.4)	1.0 (0.3 - 1.9)
30-day mortality (%)	2.9 (0.8 - 5.1)	3.6 (1.6 - 6.2)	4.1 (2.4 – 6.3)	3.8 (2.3 - 6.0)	3.8 (2.5 - 5.9)
Any rehospitalization (%)	11.6 (6.8 - 13.7)	12.7 (8.7 - 15.7)	12.6 (10.7 - 15.2)	12.8 (10.3 - 15.3)	12.6 (10.4 - 14.6)
Any non-elective rehospitalization (%)	9.9 (5.6 - 12.6)	10.8 (7.0 - 12.7)	10.9 (9.3 - 12.8)	11.2 (9.0 - 14.0)	11.0 (9.1 - 13.3)
Non-elective inpatient rehospitalization (%)	8.4 (5.1 - 11.2)	8.5 (5.8 - 10.7)	8.3 (6.2 - 11.4)	8.2 (6.2 - 9.8)	8.3 (6.2 - 9.8)
Non-elective observation rehospitalization (%)	1.8 (0.4 - 2.5)	2.9 (1.1 - 4.7)	3.2 (1.4 - 4.4)	3.7 (1.8 - 5.7)	3.3 (2.0 - 4.5)
30-day post-discharge mortality (%)	2.4 (0.5 - 4.0)	3.0 (1.2 - 5.3)	3.2 (2.3 - 4.6)	3.0 (1.8 - 4.4)	3.0 (2.0 - 4.3)
Composite outcome (%)	11.5 (5.6 - 15.5)	12.8 (7.6 - 15.0)	13.1 (11.3 - 15.4)	13.3 (11.0 - 16.7)	13.1 (10.7 - 15.9)

FOOTNOTES

a Rates employ hospitalization episodes (which can include linked stays for patients who were transported) as the denominator. Numbers in parentheses always refer to the range across the 21 study hospitals. The period preceding the penalty phase of the Hospital Readmissions Reductions Program (HRRP), which took effect on 10/1/12, is divided into two epochs, with the first of these constituting the reference period for multivariate analyses and other comparisons. The period after the penalty phase also is divided into two epochs, with the first of these (through the end of 2014) matching that of the study of Gupta et al. and the second the remaining years.

b Refers to patients whose linked hospitalization episode began at a hospital not owned by Kaiser Foundation Hospitals, Inc. These hospitalizations, involving 8,448 patients, had elevated inpatient (1.2%) and 30-day (4.2%) mortality, compared to 1.0% and 3.8% in the rest of the KPNC OBS cohort. Since we lacked information on their initial illness severity (the most important component of our mortality risk adjustment model) they are not included in inpatient and 30-day mortality analyses but are included in rehospitalization analyses, where admission illness severity plays a smaller role. See Escobar et al., 2008, 2013 and 2015, for details.

c The HEDIS membership definition restricts the denominator to patients with continuous health plan membership in the 12 months preceding and the 30 days following hospital discharge, with a maximum gap in coverage of 45 days in the preceding 12 months. The public reporting definition only includes patients meeting HEDIS membership criteria and excludes OBS hospitalizations and INP hospitalizations with length of stay < 24 hours.

d Charlson score was calculated using the methodology of Deyo et al. (1992).

e The COmorbidity Point Score, version 2 (COPS2) is assigned based on all diagnoses incurred by a patient in the 12 months preceding the index hospitalization. The univariate relationship of COPS2 with 30-day mortality is as follows: 0-39, 1.7%; 40-64, 5.2%; 65+, 9.0%. The Laboratory-based Acute Physiology Score, version 2 (LAPS2) is assigned based on a patient's worst vital signs, pulse oximetry, neurological status, and 16 laboratory test results in the preceding 24 (discharge LAPS2) or 72 hours (admission LAPS2). The univariate relationship of an admission LAPS2 with 30-day mortality is as follows: 0-59, 1.0%; 60-109, 5.0%; 110+, 13.7%; for LAPS2dc, the relationship is 0-59, 2.2%; 60-109, 8.1%; 110+, 20.5%.

f Refers to disposition among patients discharged alive from the hospital. SNF = skilled nursing facility. Note that hospice referral is independent of discharge disposition.

©2019 Escobar GJ et al. *JAMA Network Open*

g Transports-in (footnote b) are excluded from inpatient and 30-day mortality. Only patients who survived to discharge are included in the post-discharge outcomes. Non-elective rehospitalizations are those that began in the ED, were for an ambulatory care sensitive condition, and/or had a LAPS2 ≥ 60, as described in Escobar et al. (2015). Composite outcome = non-elective rehospitalization or death within 30 days after discharge.

**Table S1A-7: Patients under 65 years of age[a]**					
TIME PERIOD	*6/10-5/11*	*6/11-9/12*	*10/12-12/14*	*1/15-12/17*	*TOTAL*
	Before HRRP penalty phase		***HRRP penalties in effect***		
N (hospitalizations)	81,779	106,151	171,103	251,642	610,675
N (patients)	62,959	79,807	120,166	168,126	363,284
Inpatient (INP) (%)[b]	91.3 (82.9 - 95.7)	88.3 (81.4 - 94.4)	85.0 (73.4 - 91.8)	80.3 (61.1 - 89.7)	84.5 (74.0 - 90.9)
Observation (OBS) (%)	8.7 (4.3 - 17.1)	11.7 (5.6 - 18.6)	15.0 (8.2 - 26.6)	19.7 (10.3 - 38.9)	15.5 (9.1 - 26.0)
Inpatient stay < 24 hours	8.5 (5.4 - 10.8)	7.5 (5.0 - 10.0)	6.0 (4.2 - 8.4)	5.8 (3.0 - 8.8)	6.5 (4.2 - 8.6)
Transport-in[c]	4.2 (1.4 - 7.5)	4.3 (1.8 - 7.7)	4.1 (1.5 - 8.3)	4.3 (0.3 - 7.9)	4.2 (1.1 - 7.8)
Age (mean)	49.1 (46.6 - 51.8)	48.9 (47.0 - 51.2)	49.2 (46.8 - 51.3)	49.3 (48.0 - 51.8)	49.2 (47.5 - 51.5)
Sex (% male)	46.2 (36.6 - 53.3)	46.7 (38.0 - 54.0)	47.9 (38.7 - 56.3)	49.7 (44.6 - 55.4)	48.2 (41.0 - 55.1)
Kaiser Foundation Health Plan, Inc. member (%)	92.0 (80.3 - 96.7)	91.7 (77.8 - 96.7)	90.8 (78.8 - 96.7)	88.7 (59.6 - 95.8)	90.3 (72.8 - 96.3)
Met strict HEDIS membership definition[d] (%)	79.3 (69.6 - 84.7)	79.5 (67.5 - 86.1)	77.5 (66.2 - 84.3)	74.4 (49.1 - 81.6)	76.8 (61.3 - 83.7)
Met public reporting definition[d] (%)	66.1 (56.7 - 76.0)	64.8 (52.7 - 76.1)	61.7 (50.7 - 69.3)	55.5 (36.2 - 64.5)	60.3 (46.7 - 68.2)
Hospitalization via emergency department (%)	59.4 (43.0 - 83.0)	60.3 (44.5 - 76.8)	62.3 (44.0 - 77.4)	66.1 (45.9 - 77.2)	63.1 (44.7 - 73.9)
Charlson score[e] (median)	1.0 (0.0 - 1.0)	1.0 (0.0 - 1.0)	1.0 (0.0 - 1.0)	1.0 (1.0 - 1.0)	1.0 (1.0 - 1.0)
Charlson score ≥ 4 (%)	17.4 (12.6 - 22.1)	17.8 (12.7 - 24.5)	19.9 (13.7 - 28.0)	22.6 (16.7 - 27.4)	20.3 (15.1 - 26.4)
COPS2[f] (mean)	28.1 (24.5 - 35.7)	29.3 (24.5 - 37.6)	31.9 (25.0 - 41.4)	33.3 (24.6 - 38.6)	31.5 (25.2 - 38.8)
COPS2 ≥ 65 (%)	13.4 (10.6 - 18.7)	14.2 (10.4 - 21.0)	16.1 (10.3 - 23.2)	17.2 (10.7 - 21.2)	15.8 (10.7 - 21.4)
Admission LAPS2[f] (mean)	43.9 (36.8 - 54.9)	44.1 (36.5 - 52.3)	46.2 (37.8 - 55.1)	48.9 (39.7 - 57.1)	46.7 (38.2 - 53.9)
Discharge LAPS2 (mean)	38.6 (34.7 - 44.1)	38.3 (35.2 - 42.7)	38.1 (34.8 - 42.8)	39.0 (33.2 - 44.8)	38.6 (34.7 - 42.7)
LAPS2 ≥ 110 (%)	5.0 (3.0 - 7.9)	5.3 (2.9 - 7.8)	6.0 (3.3 - 8.6)	6.8 (3.8 - 10.0)	6.1 (3.4 - 8.5)
Full code at discharge (%)	97.1 (95.3 - 98.0)	97.0 (95.7 - 98.6)	97.1 (95.9 - 98.4)	97.2 (96.1 - 98.2)	97.1 (96.0 - 97.9)
Length of stay (days) (mean)	4.7 (3.9 - 5.3)	4.7 (3.7 - 5.4)	4.7 (3.5 - 5.4)	4.6 (3.7 - 5.2)	4.7 (3.7 - 5.3)

©2019 Escobar GJ et al. *JAMA Network Open*

Table S1A-7: Patients under 65 years of age[a]					
TIME PERIOD	*6/10-5/11*	*6/11-9/12*	*10/12-12/14*	*1/15-12/17*	*TOTAL*
	Before HRRP penalty phase		***HRRP penalties in effect***		
Discharge disposition[g] (%)					
Regular Discharge (%)	87.2 (83.0 - 94.0)	86.2 (82.3 - 93.9)	84.9 (81.1 - 94.3)	84.4 (68.1 - 95.7)	85.2 (77.0 - 94.8)
Home Health (%)	8.8 (3.1 - 13.7)	9.4 (3.4 - 13.2)	11.0 (2.8 - 15.6)	12.1 (2.5 - 24.8)	10.9 (2.8 - 15.4)
Regular SNF (%)	3.5 (1.9 - 6.7)	3.9 (2.1 - 7.1)	3.6 (2.1 - 7.1)	3.2 (1.6 - 6.3)	3.5 (1.9 - 6.5)
Custodial SNF (%)	0.4 (0.1 - 1.0)	0.5 (0.1 - 1.4)	0.5 (0.2 - 1.3)	0.3 (0.0 - 0.8)	0.4 (0.1 - 1.0)
Hospice referral (%)	0.7 (0.3 - 1.0)	0.7 (0.4 - 1.4)	0.7 (0.5 - 1.1)	0.7 (0.3 - 1.0)	0.7 (0.5 - 1.0)
Outcomes[h]					
Inpatient mortality (%)	1.3 (0.8 - 2.4)	1.3 (0.9 - 1.8)	1.4 (0.9 - 2.0)	1.4 (0.8 - 2.0)	1.4 (0.9 - 1.8)
30-day mortality (%)	2.3 (1.6 – 3.6)	2.4 (1.6 - 3.2)	2.4 (1.6 - 3.4)	2.5 (1.4 – 3.5)	2.5 (1.6 - 3.1)
Any rehospitalization (%)	12.3 (8.7 - 15.5)	12.0 (9.0 - 15.3)	12.2 (9.4 - 14.9)	12.6 (10.8 - 14.6)	12.3 (10.4 - 14.8)
Any non-elective rehospitalization (%)	9.5 (7.1 - 13.1)	9.5 (7.1 - 13.0)	9.7 (7.9 - 12.7)	10.2 (7.6 - 13.0)	9.8 (8.0 - 12.9)
Non-elective inpatient rehospitalization (%)	8.8 (6.4 - 12.0)	8.3 (5.8 - 11.9)	8.3 (6.0 - 10.6)	8.4 (6.5 - 10.7)	8.4 (6.8 - 10.7)
Non-elective observation rehospitalization (%)	1.0 (0.5 - 1.9)	1.5 (0.5 - 2.7)	1.8 (1.1 - 2.8)	2.4 (1.4 - 4.0)	1.9 (1.1 - 2.9)
30-day post-discharge mortality (%)	1.4 (1.0 - 2.0)	1.4 (0.8 - 2.1)	1.4 (0.8 - 1.9)	1.5 (0.8 - 2.0)	1.4 (1.0 – 1.9)
Composite outcome (%)	10.4 (8.0 - 14.3)	10.4 (7.7 - 14.1)	10.6 (8.5 - 13.8)	11.0 (8.4 - 14.1)	10.7 (8.7 - 13.9)

FOOTNOTES

a Rates employ hospitalization episodes (which can include linked stays for patients who were transported) as the denominator. Numbers in parentheses always refer to the range across the 21 study hospitals. The period preceding the penalty phase of the Hospital Readmissions Reductions Program (HRRP), which took effect on 10/1/12, is divided into two epochs, with the first of these constituting the reference period for multivariate analyses and other comparisons. The period after the penalty phase also is divided into two epochs, with the first of these (through the end of 2014) matching that of the study of Gupta et al. and the second the remaining years.

b Hospital episodes where patients transitioned from OBS to INP status are classified as INP.

c Refers to patients whose linked hospitalization episode began at a hospital not owned by Kaiser Foundation Hospitals, Inc. These hospitalizations, involving 23,051 patients, had elevated inpatient (2.5%) and 30-day (3.5%) mortality, compared to 1.4% and 2.5% in the rest of the KPNC under 65 cohort. Since we lacked information on their initial illness severity (the most important component of our mortality risk adjustment model) they are not included in inpatient and 30-day mortality analyses but are included in rehospitalization analyses, where admission illness severity plays a smaller role. See Escobar et al., 2008, 2013 and 2015, for details.

©2019 Escobar GJ et al. *JAMA Network Open*

d The HEDIS membership definition restricts the denominator to patients with continuous health plan membership in the 12 months preceding and the 30 days following hospital discharge, with a maximum gap in coverage of 45 days in the preceding 12 months. The public reporting definition only includes patients meeting HEDIS membership criteria and excludes OBS hospitalizations and INP hospitalizations with length of stay < 24 hours.

e Charlson score was calculated using the methodology of Deyo et al. (1992).

f The COmorbidity Point Score, version 2 (COPS2) is assigned based on all diagnoses incurred by a patient in the 12 months preceding the index hospitalization. The univariate relationship of COPS2 with 30-day mortality is as follows: 0-39, 1.7%; 40-64, 5.2%; 65+, 9.0%. The Laboratory-based Acute Physiology Score, version 2 (LAPS2) is assigned based on a patient's worst vital signs, pulse oximetry, neurological status, and 16 laboratory test results in the preceding 24 (discharge LAPS2) or 72 hours (admission LAPS2). The univariate relationship of an admission LAPS2 with 30-day mortality is as follows: 0-59, 1.0%; 60-109, 5.0%; 110+, 13.7%; for LAPS2dc, the relationship is 0-59, 2.2%; 60-109, 8.1%; 110+, 20.5%.

g Refers to disposition among patients discharged alive from the hospital. SNF = skilled nursing facility. Note that hospice referral is independent of discharge disposition.

h Transports-in (footnote c) are excluded from inpatient and 30-day mortality. Only patients who survived to discharge are included in the post-discharge outcomes. Non-elective rehospitalizations are those that began in the ED, were for an ambulatory care sensitive condition, and/or had a LAPS2 ≥ 60, as described in Escobar et al. (2015). Composite outcome = non-elective rehospitalization or death within 30 days after discharge.

©2019 Escobar GJ et al. *JAMA Network Open*

Table S1A-8: Patients ≥ 65 years of age[a]					
TIME PERIOD	*6/10-5/11*	*6/11-9/12*	*10/12-12/14*	*1/15-12/17*	*TOTAL*
	Before HRRP penalty phase		***HRRP penalties in effect***		
N (hospitalizations)	93,505	124,632	219,447	335,766	773,350
N (patients)	64,579	81,826	130,831	184,691	330,667
Inpatient (INP) (%)[b]	89.9 (81.4 - 95.5)	86.1 (73.1 - 93.9)	83.1 (70.7 - 90.5)	79.0 (56.2 - 89.8)	82.6 (69.0 - 90.2)
Observation (OBS) (%)	10.1 (4.5 - 18.6)	13.9 (6.1 - 26.9)	16.9 (9.5 - 29.3)	21.0 (10.2 - 43.8)	17.4 (9.8 - 31.0)
Inpatient stay < 24 hours	5.4 (2.7 - 7.9)	4.7 (2.6 - 6.6)	3.8 (2.3 - 5.2)	3.8 (1.2 - 5.8)	4.1 (2.8 - 5.1)
Transport-in[c]	5.0 (1.0 - 9.2)	5.0 (1.4 - 10.3)	4.5 (2.3 - 9.8)	4.5 (0.4 - 9.5)	4.7 (1.7 - 9.6)
Age (mean)	78.0 (76.6 - 79.0)	77.9 (76.4 - 78.9)	77.8 (76.2 - 79.0)	77.8 (76.4 - 79.1)	77.8 (76.3 - 78.9)
Sex (% male)	45.7 (43.2 - 53.0)	46.0 (42.4 - 52.6)	46.5 (43.8 - 52.0)	47.1 (43.3 - 52.5)	46.6 (43.8 - 52.4)
Kaiser Foundation Health Plan, Inc. member (%)	97.1 (92.9 - 98.9)	97.2 (92.1 - 98.9)	96.8 (89.4 - 99.0)	95.7 (70.4 - 98.8)	96.4 (82.1 - 98.9)
Met strict HEDIS membership definition[d] (%)	84.0 (79.1 - 88.8)	84.6 (78.2 - 89.3)	83.6 (75.5 - 88.2)	82.2 (59.1 - 87.2)	83.2 (69.3 - 88.1)
Met public reporting definition[d] (%)	70.7 (58.7 - 79.2)	68.7 (57.0 - 78.6)	66.0 (54.2 - 74.1)	61.1 (43.7 - 71.9)	64.9 (50.4 - 73.2)
Hospitalization via emergency department (%)	73.1 (57.8 - 89.7)	73.1 (59.9 - 90.9)	74.5 (59.1 - 90.7)	75.7 (60.4 - 96.1)	74.6 (59.6 - 93.1)
Charlson score[e] (median)	3.0 (2.0 - 4.0)	3.0 (2.0 - 4.0)	3.0 (2.0 - 4.0)	4.0 (3.0 - 4.0)	3.0 (2.0 - 4.0)
Charlson score ≥ 4 (%)	38.8 (31.3 - 50.4)	41.0 (32.0 - 53.9)	43.8 (34.5 - 52.6)	50.1 (39.8 - 58.3)	45.5 (35.8 - 54.8)
COPS2[f] (mean)	45.3 (40.2 - 52.2)	49.1 (41.7 - 56.9)	55.8 (48.2 - 64.9)	58.8 (51.0 - 66.6)	54.7 (47.2 - 61.8)
COPS2 ≥ 65 (%)	26.8 (21.9 - 34.2)	29.5 (24.1 - 37.6)	34.9 (28.5 - 43.3)	37.5 (30.6 - 45.5)	34.2 (27.8 - 41.7)
Admission LAPS2[f] (mean)	65.3 (55.1 - 81.2)	65.7 (54.5 - 81.0)	66.8 (53.9 - 80.2)	67.9 (55.7 - 81.8)	66.9 (54.9 - 81.2)
Discharge LAPS2 (mean)	53.1 (47.0 - 59.9)	53.3 (49.2 - 59.3)	52.4 (47.9 - 57.3)	52.7 (46.7 - 58.2)	52.7 (47.9 - 58.0)
LAPS2 ≥ 110 (%)	15.0 (11.2 - 23.9)	15.6 (10.0 - 22.9)	16.2 (9.7 - 22.4)	16.9 (10.3 - 22.5)	16.3 (10.5 - 22.7)
Full code at discharge (%)	74.3 (66.1 - 80.9)	74.3 (67.0 - 83.4)	73.9 (67.5 - 82.3)	74.4 (67.9 - 82.9)	74.2 (67.7 - 82.4)
Length of stay (days) (mean)	5.2 (4.5 - 6.0)	5.2 (4.3 - 5.9)	5.0 (4.0 - 5.6)	4.9 (4.1 - 5.7)	5.0 (4.2 - 5.7)
Discharge disposition[g] (%)					
Regular Discharge (%)	65.6 (58.5 - 74.6)	64.1 (56.5 - 75.1)	62.5 (52.9 - 74.8)	62.0 (42.4 - 79.2)	62.9 (50.8 - 76.9)
Home Health (%)	15.5 (11.2 - 24.1)	16.8 (10.2 - 25.5)	19.9 (12.3 - 30.2)	22.3 (10.3 - 35.5)	19.9 (11.2 - 29.6)

©2019 Escobar GJ et al. *JAMA Network Open*

Table S1A-8: Patients ≥ 65 years of age[a]					
TIME PERIOD	*6/10-5/11*	*6/11-9/12*	*10/12-12/14*	*1/15-12/17*	*TOTAL*
	Before HRRP penalty phase		***HRRP penalties in effect***		
Regular SNF (%)	16.2 (10.8 - 24.5)	16.6 (10.9 - 24.8)	15.3 (11.0 - 22.5)	13.9 (9.1 - 18.7)	15.0 (10.2 - 21.1)
Custodial SNF (%)	2.7 (1.4 - 3.9)	2.5 (1.3 - 4.6)	2.4 (1.3 - 4.3)	1.8 (0.8 - 3.3)	2.2 (1.3 - 3.7)
Hospice referral (%)	4.1 (3.0 - 5.9)	4.0 (2.7 - 6.7)	4.1 (2.8 - 7.7)	4.1 (2.6 - 6.2)	4.1 (2.8 - 6.7)
Outcomes[h]					
Inpatient mortality (%)	4.1 (2.9 - 5.3)	3.9 (2.6 - 5.7)	3.9 (2.7 – 4.9)	3.8 (3.0 – 4.9)	3.9 (2.9 - 4.8)
30-day mortality (%)	9.0 (5.7 - 11.6)	9.0 (5.5 - 11.2)	8.9 (5.9 - 11.2)	8.8 (6.2 - 11.0)	8.9 (6.0 - 10.9)
Any rehospitalization (%)	16.3 (13.2 - 19.0)	16.2 (12.6 - 18.8)	15.7 (13.4 - 19.3)	16.2 (13.1 - 18.9)	16.1 (13.5 - 18.9)
Any non-elective rehospitalization (%)	14.2 (11.1 - 17.6)	14.2 (10.6 - 17.0)	13.9 (11.4 - 17.5)	14.4 (11.1 - 17.8)	14.2 (11.2 - 17.2)
Non-elective inpatient rehospitalization (%)	13.1 (10.0 - 15.9)	12.5 (9.0 - 15.2)	11.8 (8.5 - 14.2)	11.8 (8.4 - 15.8)	12.1 (8.7 - 14.9)
Non-elective observation rehospitalization (%)	1.4 (0.7 - 2.4)	2.2 (0.7 - 3.6)	2.6 (1.1 - 4.1)	3.3 (1.8 - 6.4)	2.7 (1.4 - 4.2)
30-day post-discharge mortality (%)	6.2 (3.7 - 9.1)	6.3 (4.2 - 8.3)	6.3 (4.1 - 8.2)	6.2 (4.1 - 7.7)	6.2 (4.1 - 8.0)
Composite outcome (%)	18.7 (14.8 - 23.3)	18.7 (15.3 - 22.9)	18.4 (15.7 - 23.2)	18.8 (15.4 - 23.3)	18.7 (15.6 - 22.7)

FOOTNOTES

a Rates employ hospitalization episodes (which can include linked stays for patients who were transported) as the denominator. Numbers in parentheses always refer to the range across the 21 study hospitals. The period preceding the penalty phase of the Hospital Readmissions Reductions Program (HRRP), which took effect on 10/1/12, is divided into two epochs, with the first of these constituting the reference period for multivariate analyses and other comparisons. The period after the penalty phase also is divided into two epochs, with the first of these (through the end of 2014) matching that of the study of Gupta et al. and the second the remaining years.

b Hospital episodes where patients transitioned from OBS to INP status are classified as INP.

c Refers to patients whose linked hospitalization episode began at a hospital not owned by Kaiser Foundation Hospitals, Inc. These hospitalizations, involving 29,969 patients, had elevated inpatient (6.8%) and 30-day (13.7%) mortality, compared to 3.9% and 8.9% in the rest of the KPNC 65+ cohort. Since we lacked information on their initial illness severity (the most important component of our mortality risk adjustment model) they are not included in inpatient and 30-day mortality analyses but are included in rehospitalization analyses, where admission illness severity plays a smaller role. See Escobar et al., 2008, 2013 and 2015, for details.

d The HEDIS membership definition restricts the denominator to patients with continuous health plan membership in the 12 months preceding and the 30 days following hospital discharge, with a maximum gap in coverage of 45 days in the preceding 12 months. The public reporting definition only includes patients meeting HEDIS membership criteria and excludes OBS hospitalizations and INP hospitalizations with length of stay < 24 hours.

e Charlson score was calculated using the methodology of Deyo et al. (1992).

©2019 Escobar GJ et al. *JAMA Network Open*

f The COmorbidity Point Score, version 2 (COPS2) is assigned based on all diagnoses incurred by a patient in the 12 months preceding the index hospitalization. The univariate relationship of COPS2 with 30-day mortality is as follows: 0-39, 1.7%; 40-64, 5.2%; 65+, 9.0%. The Laboratory-based Acute Physiology Score, version 2 (LAPS2) is assigned based on a patient's worst vital signs, pulse oximetry, neurological status, and 16 laboratory test results in the preceding 24 (discharge LAPS2) or 72 hours (admission LAPS2). The univariate relationship of an admission LAPS2 with 30-day mortality is as follows: 0-59, 1.0%; 60-109, 5.0%; 110+, 13.7%; for LAPS2dc, the relationship is 0-59, 2.2%; 60-109, 8.1%; 110+, 20.5%.

g Refers to disposition among patients discharged alive from the hospital. SNF = skilled nursing facility. Note that hospice referral is independent of discharge disposition.

h Transports-in (footnote c) are excluded from inpatient and 30-day mortality. Only patients who survived to discharge are included in the post-discharge outcomes. Non-elective rehospitalizations are those that began in the ED, were for an ambulatory care sensitive condition, and/or had a LAPS2 ≥ 60, as described in Escobar et al. (2015). Composite outcome = non-elective rehospitalization or death within 30 days after discharge.

©2019 Escobar GJ et al. *JAMA Network Open*

Table S1A-9: Proportions of very ill patients[a]						
TIME PERIOD	*6/10-5/11*	*6/11-9/12*	*10/12-12/14*	*1/15-12/17*	*TOTAL*	*P*[b]
	Before HRRP penalty phase		***HRRP penalties in effect***			
**Table 1 ALL**						
N	175,284	230,783	390,550	587,408	1,384,025	
% with COPS2 ≥ 65	20.5 (18.4 - 26.4)	22.5 (18.8 - 28.1)	26.7 (21.8 - 33.7)	28.8 (22.3 - 33.0)	26.1 (21.2 - 31.5)	<.0001
% with LAPS2 ≥ 110	10.3 (7.4 – 14.7)	10.8 (7.1 – 15.9)	11.7 (6.9 – 15.8)	12.5 (8.3 - 16.6)	11.7 (7.6 - 15.5)	<.0001
% with Charlson ≥ 4	28.8 (24.6 - 35.0)	30.4 (25.0 - 37.6)	33.3 (27.4 - 40.4)	38.4 (31.9 - 43.4)	34.4 (28.7 - 40.0)	<.0001
**APDX Table 1A-1 CAP**						
N	4,379	3,859	5,813	9,894	23,945	
% with COPS2 ≥ 65	32.5 (26.7 - 39.6)	35.6 (25.9 - 44.3)	44.1 (37.6 - 52.7)	44.5 (32.6 - 50.7)	40.8 (32.0 - 47.4)	<.0001
% with LAPS2 ≥ 110	19.1 (10.4 - 27.3)	18.1 (10.1 - 23.8)	17.4 (6.0 - 23.5)	17.5 (10.4 - 25.1)	17.9 (11.5 - 23.2)	0.0980
% with Charlson ≥ 4	39.4 (29.4 - 51.7)	39.8 (30.2 - 52.6)	46.1 (36.6 - 55.8)	51.1 (44.2 - 55.9)	45.9 (39.0 - 50.6)	<.0001
**APDX Table 1A-2 CHF**						
N	6,018	7,501	14,077	17,511	45,107	
% with COPS2 ≥ 65	49.0 (39.9 - 56.0)	55.0 (47.0 - 61.4)	59.1 (50.7 - 69.8)	58.2 (43.4 - 64.8)	56.7 (48.7 - 62.7)	<.0001
% with LAPS2 ≥ 110	20.0 (10.9 - 26.6)	19.9 (12.8 - 33.3)	20.8 (12.9 - 30.4)	19.8 (14.3 - 28.9)	20.2 (15.2 - 27.7)	0.2281
% with Charlson ≥ 4	61.4 (48.0 - 72.1)	65.4 (57.0 - 77.2)	66.5 (59.3 - 75.1)	67.8 (58.5 - 78.0)	66.1 (59.3 - 76.0)	<.0001
**APDX Table 1A-3 AMI**						
N	4,194	5,471	10,131	15,684	35,480	
% with COPS2 ≥ 65	16.7 (9.2 - 31.7)	16.7 (11.4 - 31.1)	20.6 (15.1 - 36.4)	20.3 (11.7 - 50.0)	19.4 (15.2 - 33.9)	<.0001
% with LAPS2 ≥ 110	14.5 (5.4 - 24.0)	13.5 (6.3 - 25.4)	14.3 (9.3 - 22.1)	12.6 (8.0 - 37.5)	13.5 (9.5 - 21.1)	0.0005
% with Charlson ≥ 4	32.8 (20.4 - 47.4)	32.9 (22.2 - 57.8)	35.8 (28.1 - 52.3)	36.9 (26.2 - 61.8)	35.5 (28.1 - 51.5)	<.0001
**APDX Table 1A-4 No CAP-CHF-AMI**						
N	160,693	213,952	360,529	544,319	1,279,493	
% with COPS2 ≥ 65	19.3 (17.4 - 25.0)	21.2 (18.0 - 27.0)	25.3 (20.6 - 32.2)	27.8 (21.4 - 32.1)	24.9 (20.1 - 30.3)	<.0001
% with LAPS2 ≥ 110	9.6 (6.7 - 14.4)	10.3 (6.7 - 14.4)	11.2 (6.6 - 15.4)	12.2 (7.8 - 16.5)	11.3 (7.1 - 15.4)	<.0001

©2019 Escobar GJ et al. *JAMA Network Open*

Table S1A-9: Proportions of very ill patients[a]						
TIME PERIOD	*6/10-5/11*	*6/11-9/12*	*10/12-12/14*	*1/15-12/17*	*TOTAL*	*P*[b]
	Before HRRP penalty phase		***HRRP penalties in effect***			
% with Charlson ≥ 4	27.2 (23.6 - 32.7)	28.9 (24.1 - 35.3)	31.7 (26.1 - 38.9)	37.2 (31.1 - 42.7)	33.0 (27.6 - 38.6)	<.0001
**APDX Table 1A-5 INP**						
N	158,787	201,104	327,950	467,193	1,155,034	
% with COPS2 ≥ 65	20.8 (18.3 - 27.1)	22.5 (18.1 - 29.0)	26.7 (21.1 - 35.5)	29.0 (22.0 - 34.0)	26.1 (21.1 - 32.0)	<.0001
% with LAPS2 ≥ 110	11.0 (7.7 - 15.9)	11.6 (7.5 - 16.8)	12.8 (7.5 - 18.5)	14.0 (9.1 - 18.9)	12.8 (8.1 - 17.6)	<.0001
% with Charlson ≥ 4	28.9 (24.9 - 35.2)	30.1 (24.9 - 36.6)	33.2 (27.3 - 41.9)	38.2 (31.6 - 44.2)	34.1 (28.5 - 40.0)	<.0001
**APDX Table 1A-6 OBS**						
N	16,497	29,679	62,600	120,215	228,991	
% with COPS2 ≥ 65	17.9 (14.4 - 21.2)	21.9 (14.8 - 29.5)	26.2 (21.4 - 31.4)	28.1 (20.2 - 34.8)	26.1 (22.3 - 31.0)	<.0001
% with LAPS2 ≥ 110	4.1 (1.5 – 7.7)	5.5 (2.3 – 9.3)	6.3 (3.6 - 8.8)	6.8 (3.8 - 9.8)	6.3 (3.7 – 8.5)	<.0001
% with Charlson ≥ 4	28.1 (22.6 - 33.7)	31.9 (25.6 - 44.1)	33.7 (28.2 - 38.3)	38.9 (33.7 - 45.6)	35.8 (30.1 - 42.3)	<.0001
**APDX Table 1A-7 < 65**						
N	81,779	106,151	171,103	251,642	610,675	
% with COPS2 ≥ 65	13.4 (10.6 - 18.7)	14.2 (10.4 - 21.0)	16.1 (10.3 - 23.2)	17.2 (10.7 - 21.2)	15.8 (10.7 - 21.4)	<.0001
% with LAPS2 ≥ 110	5.0 (3.0 - 7.9)	5.3 (2.9 – 7.9)	6.0 (3.3 - 8.6)	6.8 (3.8 - 10.0)	6.1 (3.4 - 8.5)	<.0001
% with Charlson ≥ 4	17.4 (12.6 - 22.1)	17.8 (12.7 - 24.5)	19.9 (13.7 - 28.0)	22.6 (16.7 - 27.4)	20.3 (15.1 - 26.4)	<.0001
**APDX Table 1A-8 65+**						
N	93,505	124,632	219,447	335,766	773,350	
% with COPS2 ≥ 65	26.8 (21.9 - 34.2)	29.5 (24.1 - 37.6)	34.9 (28.5 - 43.3)	37.5 (30.6 - 45.5)	34.2 (27.8 - 41.7)	<.0001
% with LAPS2 ≥ 110	15.0 (11.2 - 23.9)	15.6 (10.0 - 22.9)	16.2 (9.7 - 22.4)	16.9 (10.3 - 22.5)	16.3 (10.5 - 22.7)	<.0001
% with Charlson ≥ 4	38.8 (31.3 - 50.4)	41.0 (32.0 - 53.9)	43.8 (34.5 - 52.6)	50.1 (39.8 - 58.3)	45.5 (35.8 - 54.8)	<.0001

FOOTNOTES

a Numbers in this table show the proportion of very ill patients (using longitudinal comorbidity or severity of illness) in the entire cohort (Table 1 in the main text) or in the various patient subsets (Appendix tables 1A-1 through 1A-8). Numbers in parentheses refer to the range across the 21 study hospitals.

b P value is for comparison of proportions over time. See main text for details.

eAppendix 2. Unadjusted Outcomes

Figure 2A-1: Patients with community-acquired pneumonia (CAP)

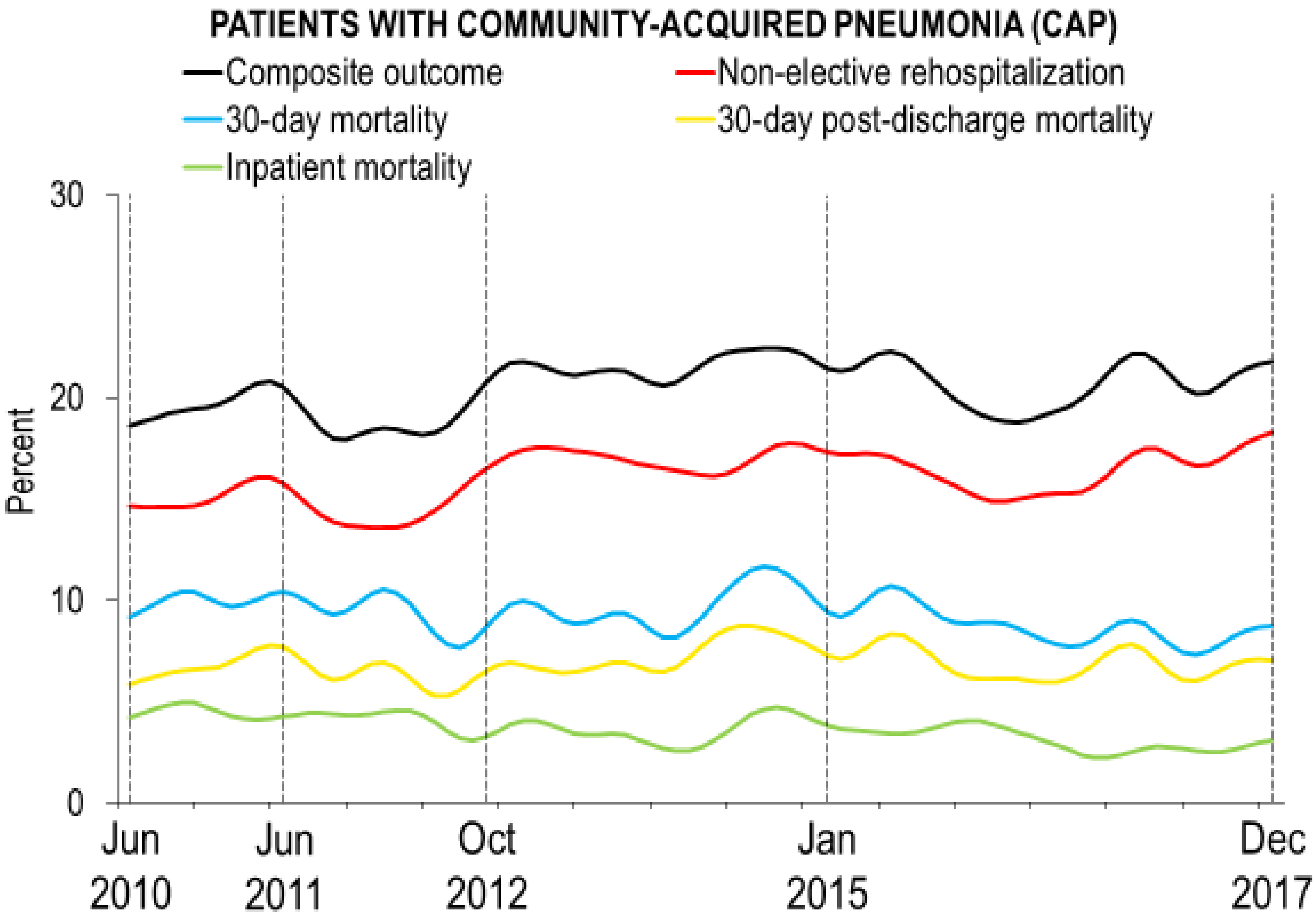

©2019 Escobar GJ et al. *JAMA Network Open*

Figure 2A-2: Patients with congestive heart failure (CHF)

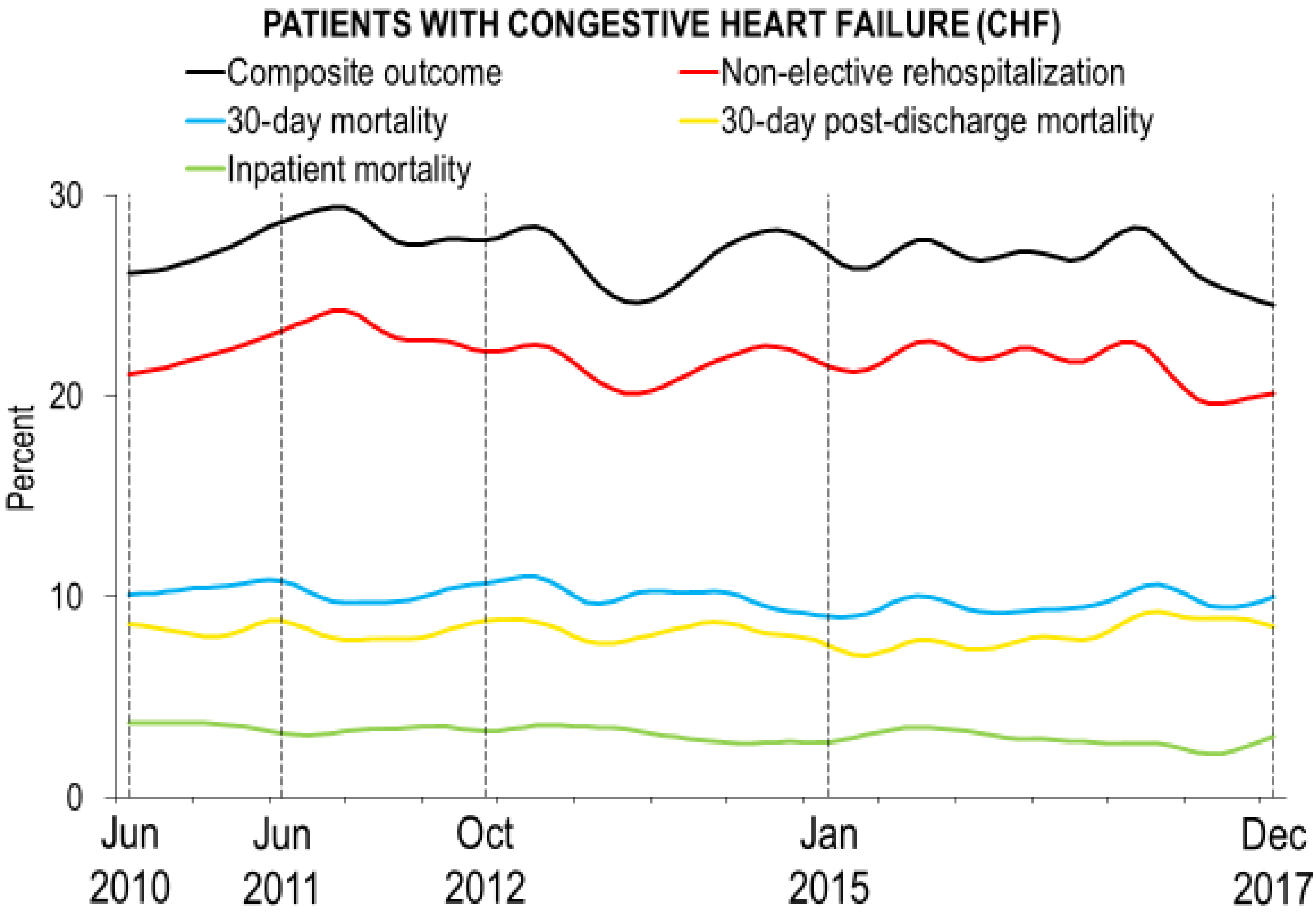

©2019 Escobar GJ et al. *JAMA Network Open*

Figure 2A-3: Patients with acute myocardial infarction (AMI)

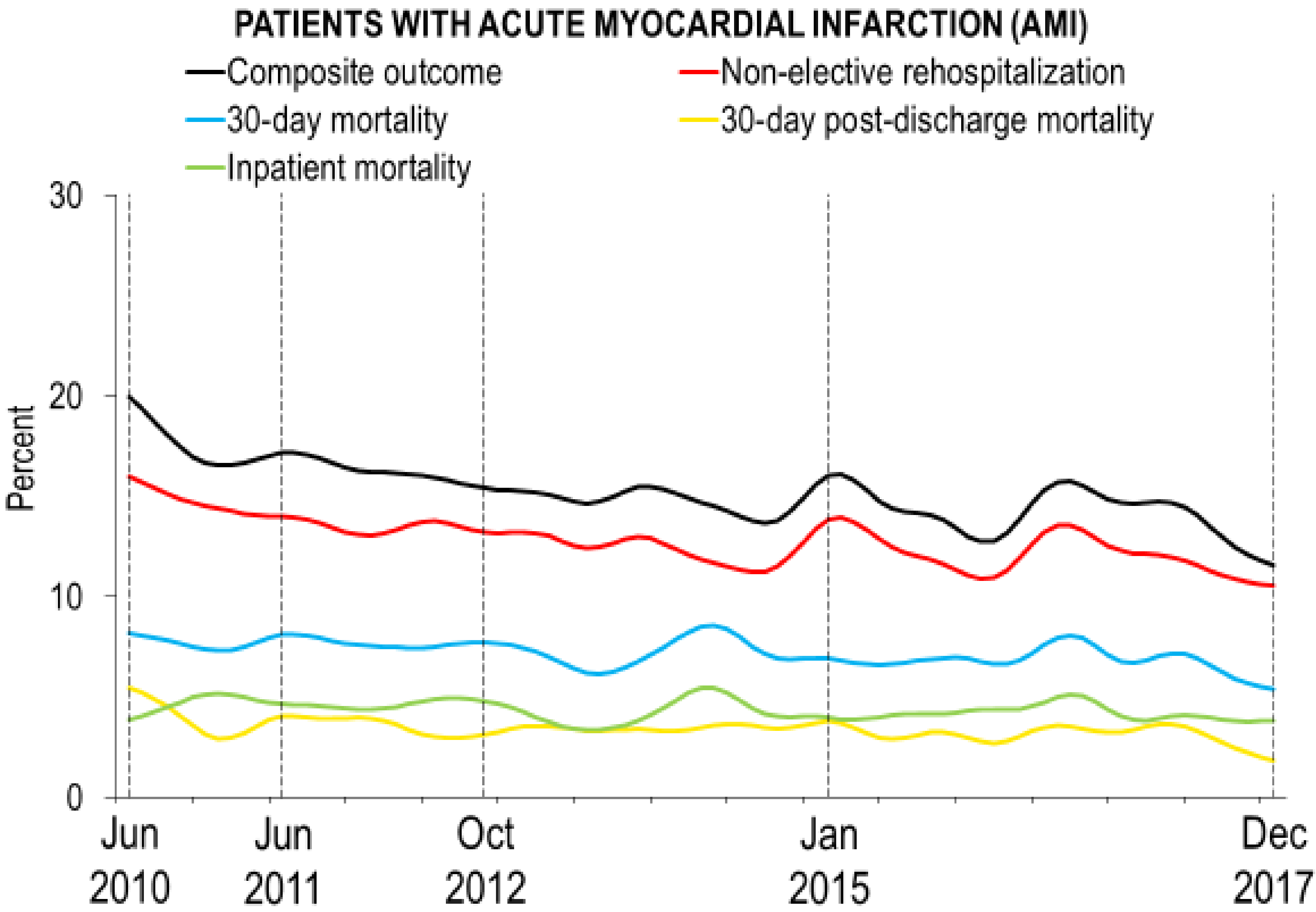

©2019 Escobar GJ et al. *JAMA Network Open*

Figure 2A-4: Patients without CAP, CHF, or AMI

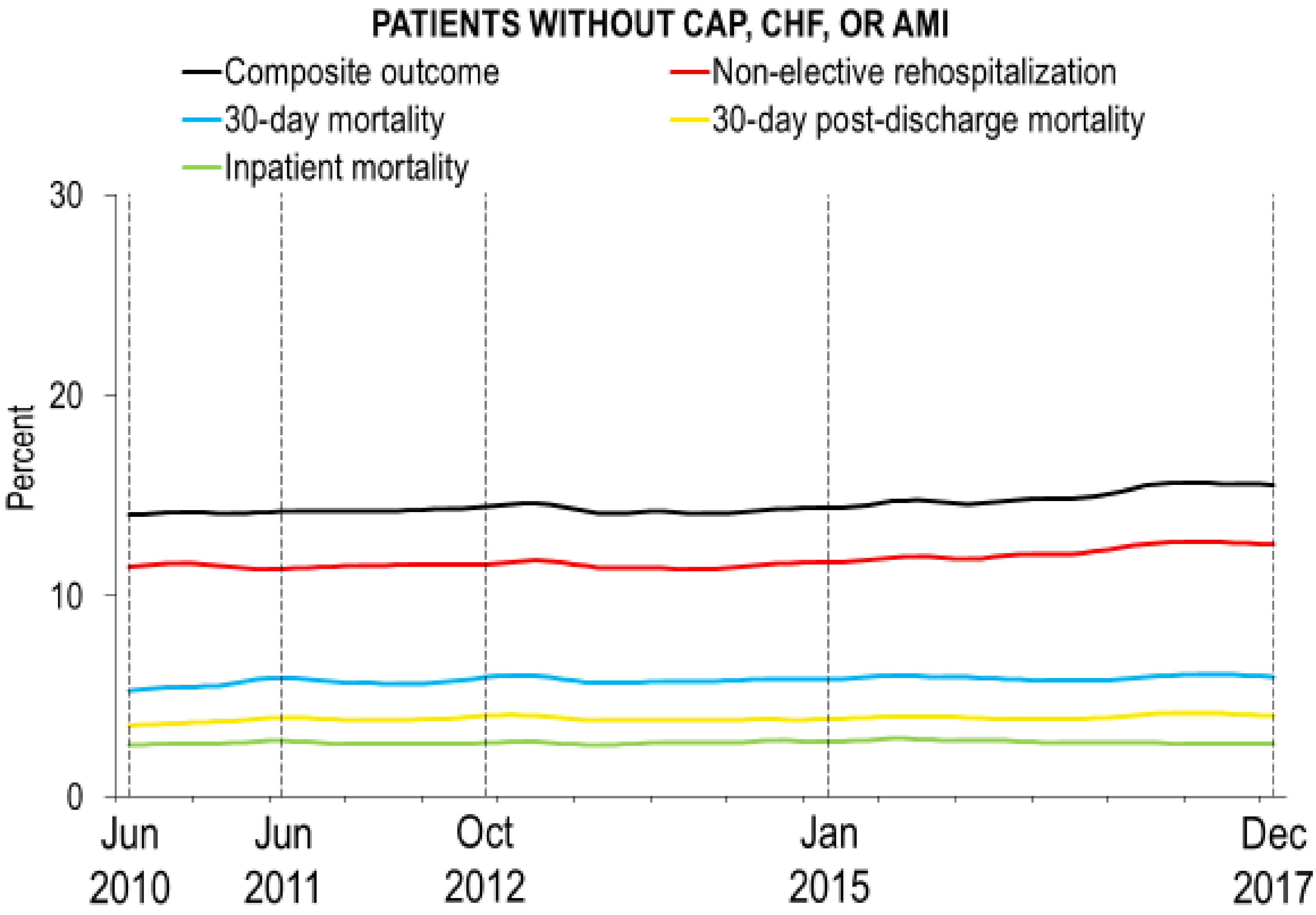

©2019 Escobar GJ et al. *JAMA Network Open*

Figure 2A-5: Inpatients only

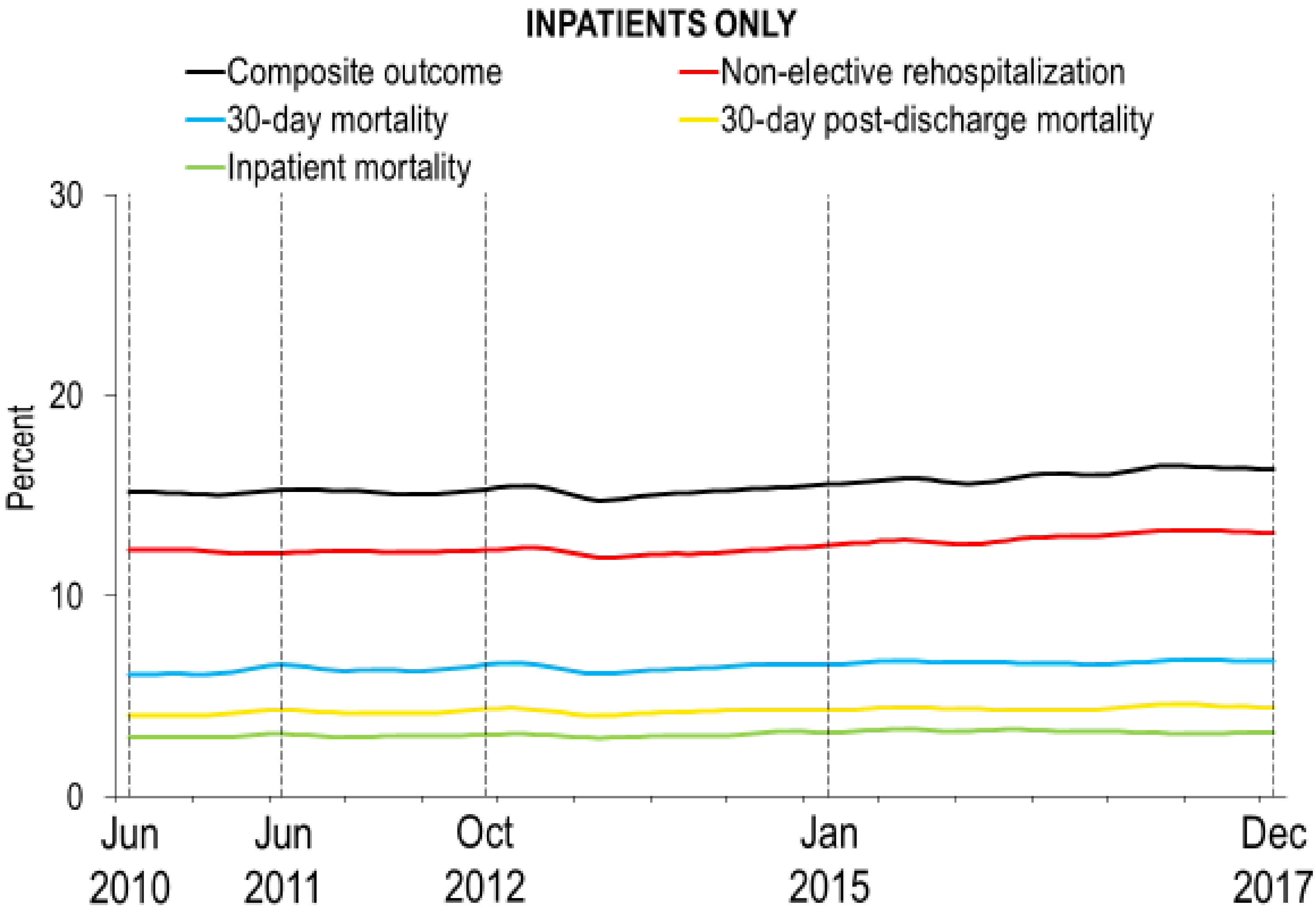

©2019 Escobar GJ et al. *JAMA Network Open*

Figure 2A-6: Observation hospitalizations only

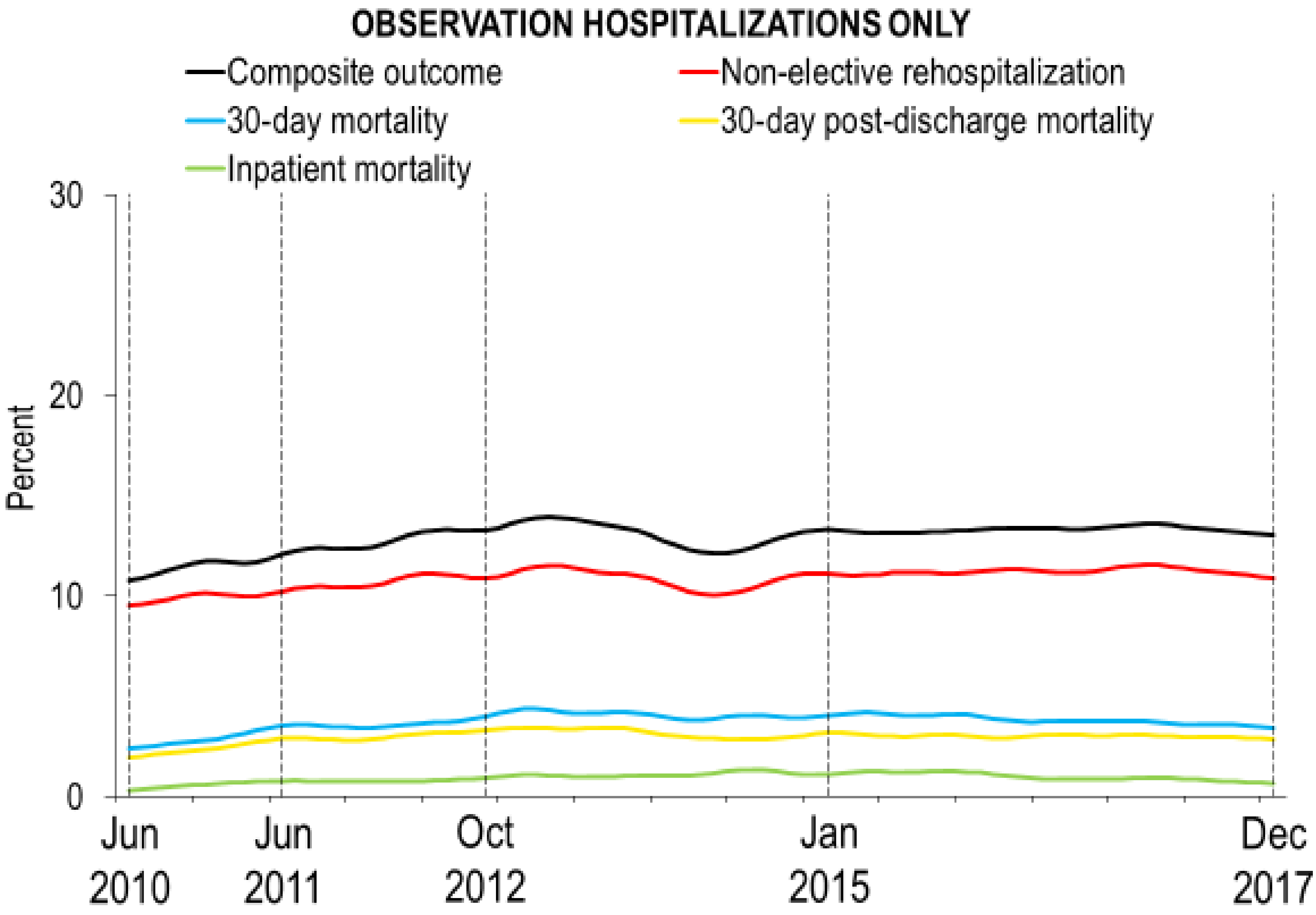

©2019 Escobar GJ et al. *JAMA Network Open*

eAppendix 3. Proportions of Different Hospitalization Types

Figure 3A-1: Patients under 65 years of age

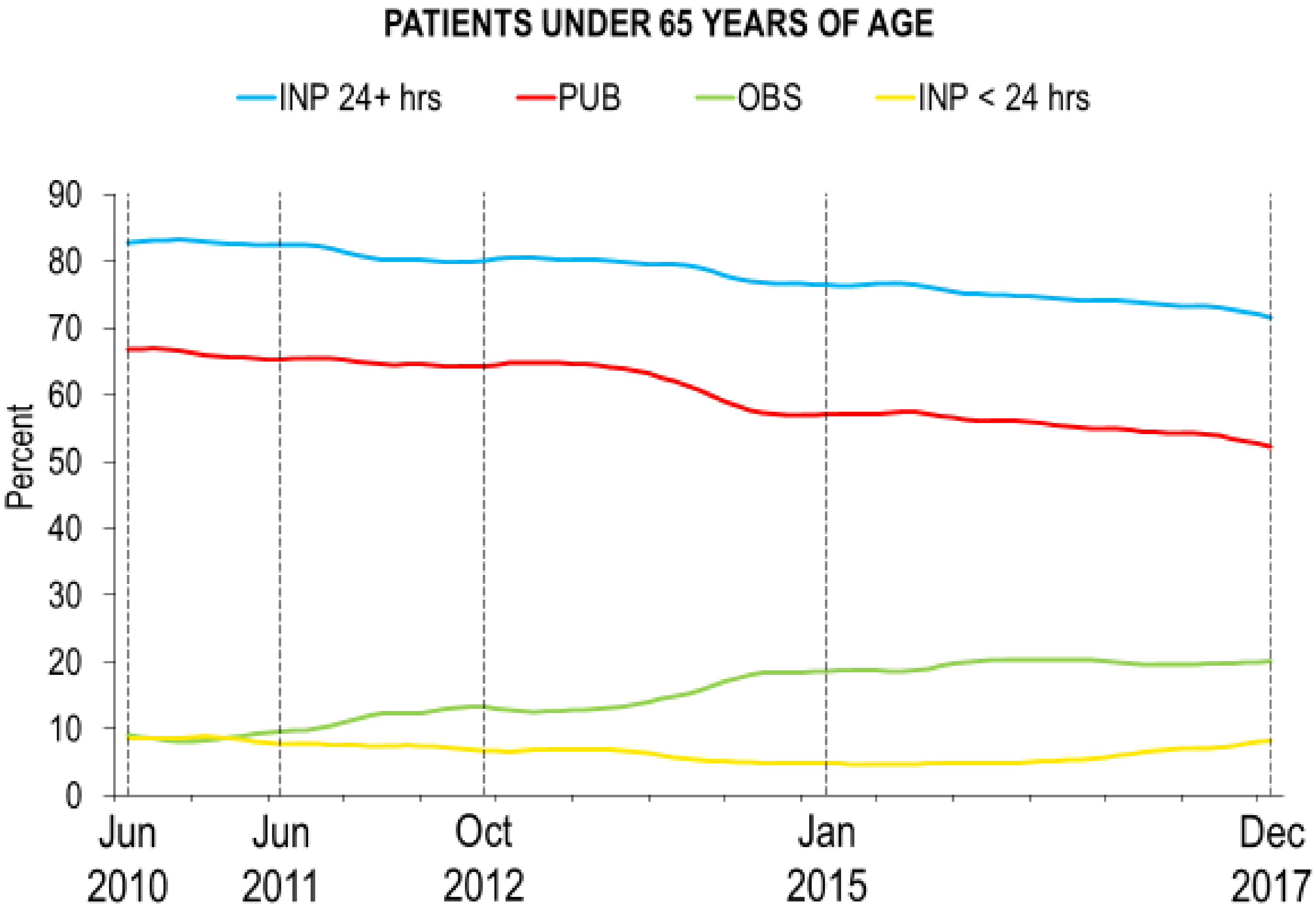

©2019 Escobar GJ et al. *JAMA Network Open*

Figure 3A-2: Patients ≥ 65 years of age

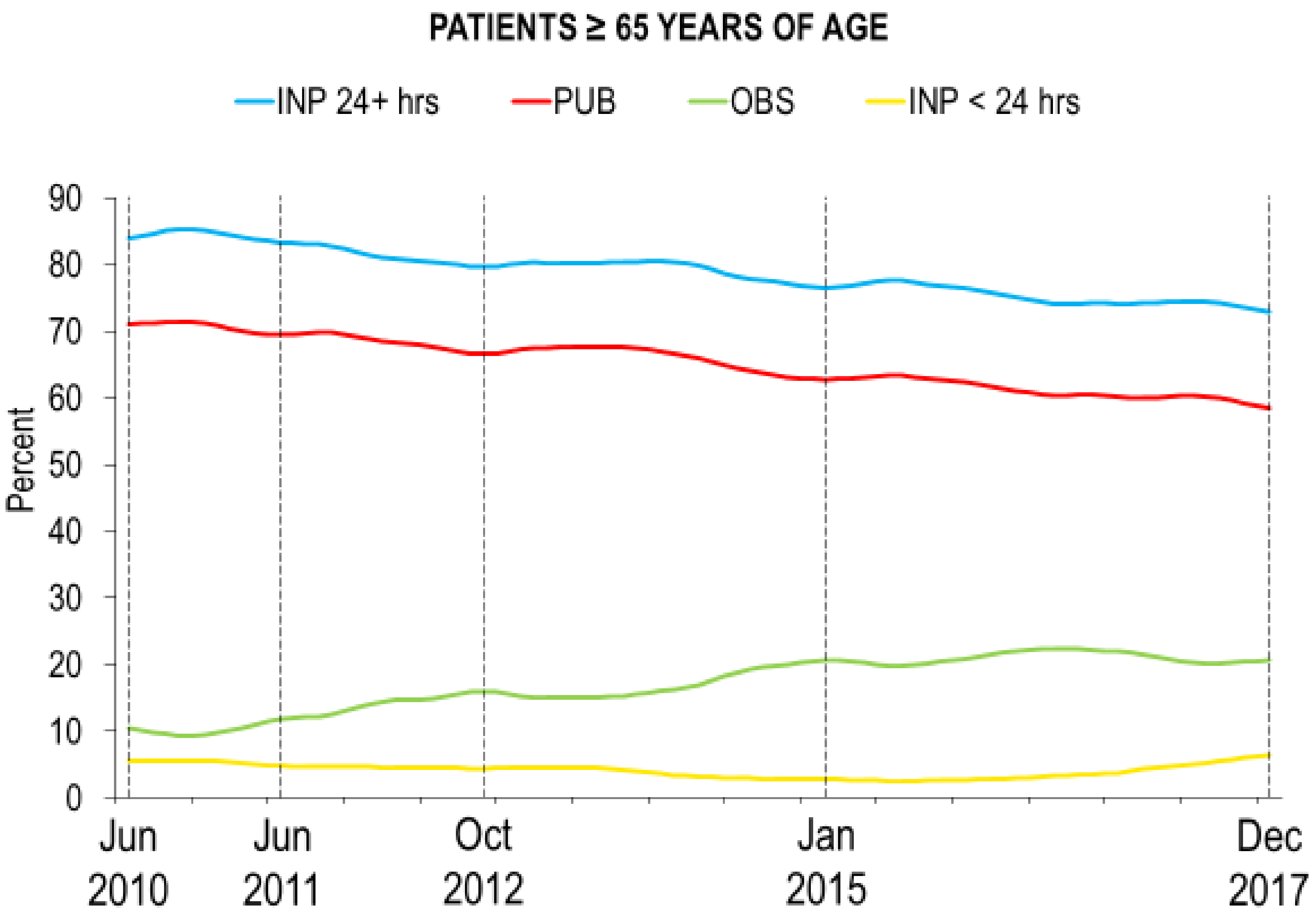

©2019 Escobar GJ et al. *JAMA Network Open*

eAppendix 4. Adjusted Outcomes

Figure 4A-1: Inpatient Mortality, All Hospitalizations

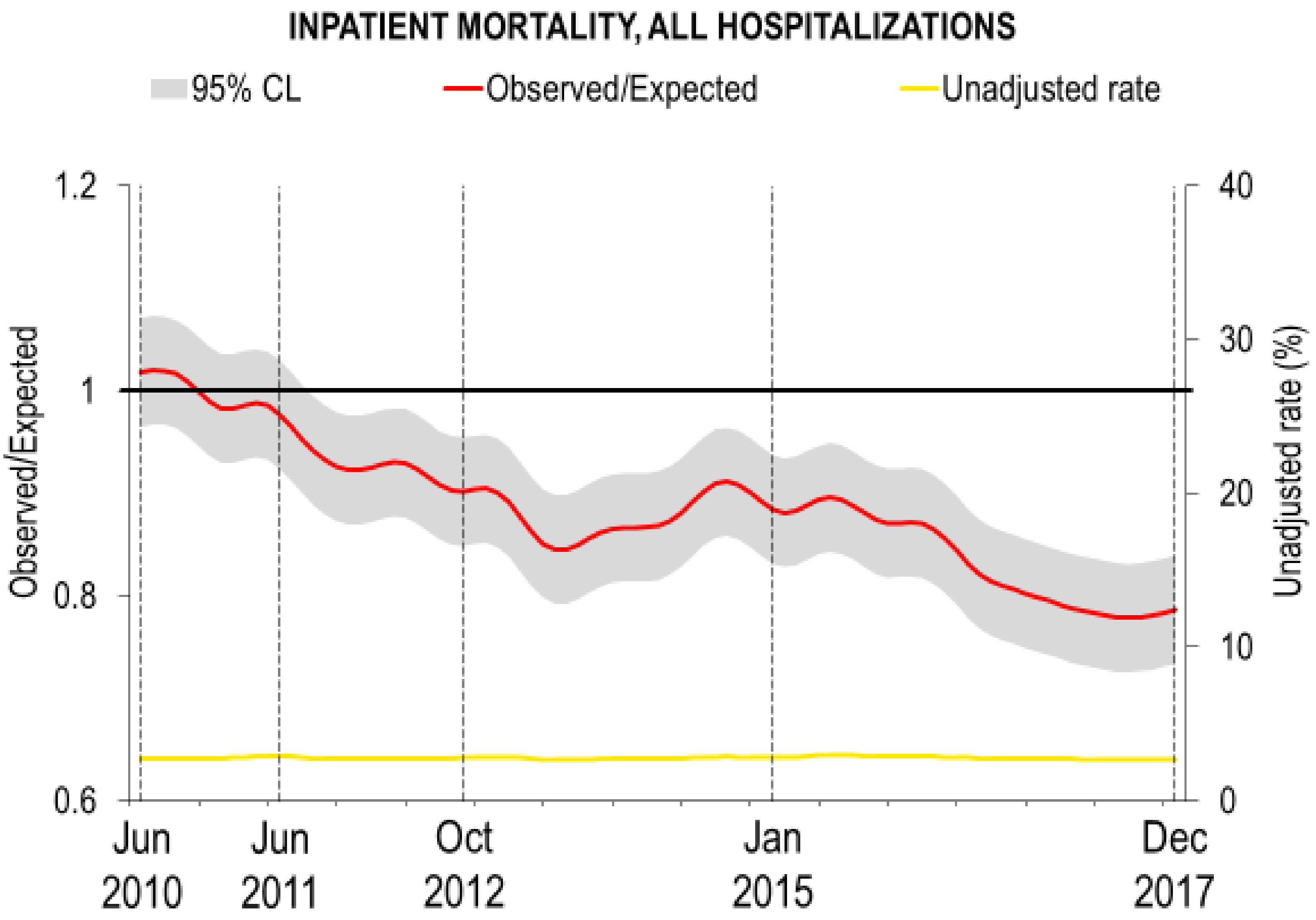

©2019 Escobar GJ et al. *JAMA Network Open*

Figure 4A-2: 30-day Mortality, All Hospitalizations

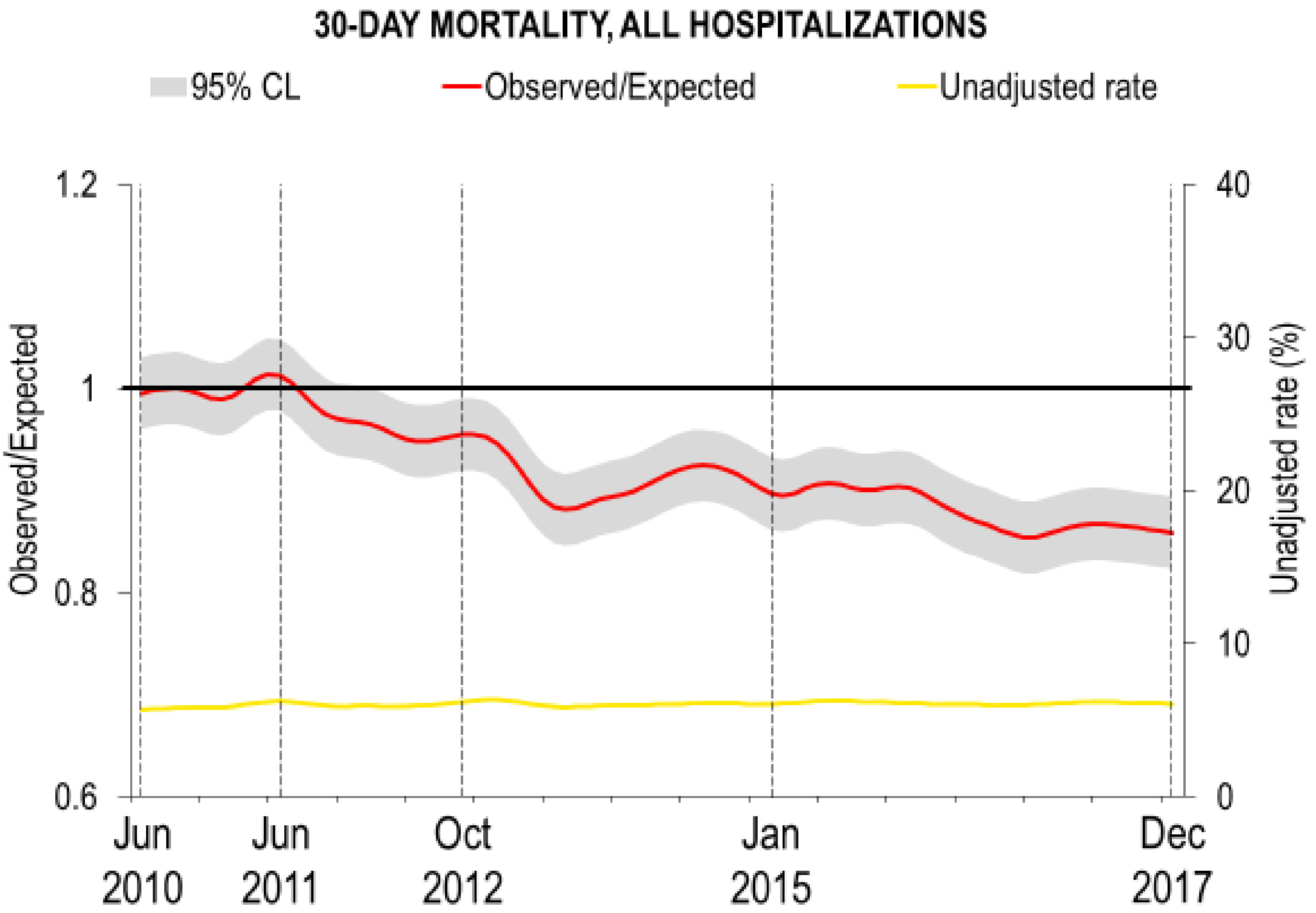

©2019 Escobar GJ et al. *JAMA Network Open*

Figure 4A-3: 30-day Composite Outcome, All Hospitalizations

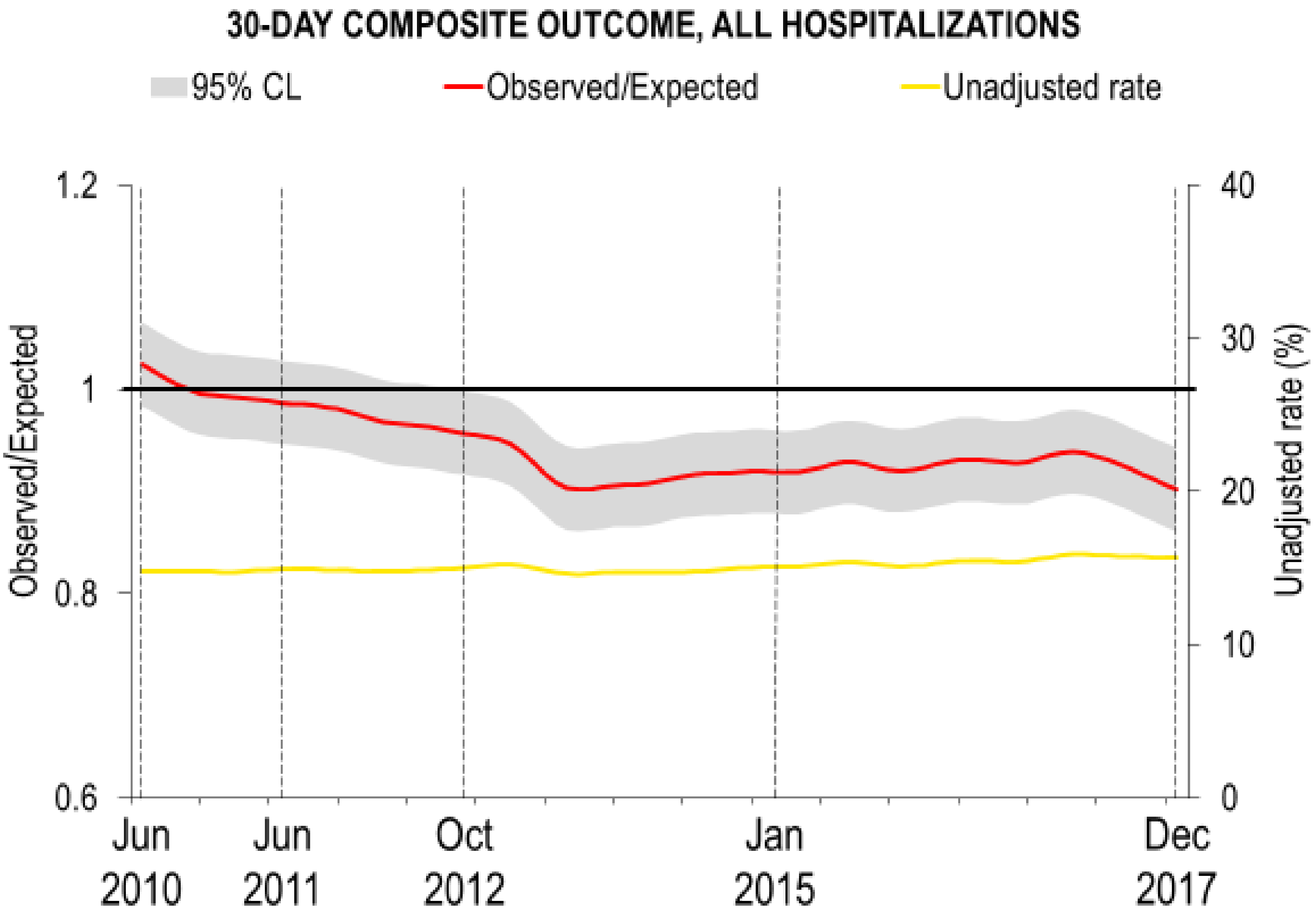

©2019 Escobar GJ et al. *JAMA Network Open*

Figure 4A-4: Non-elective Rehospitalization, Index Stays Meeting Public Reporting Definition

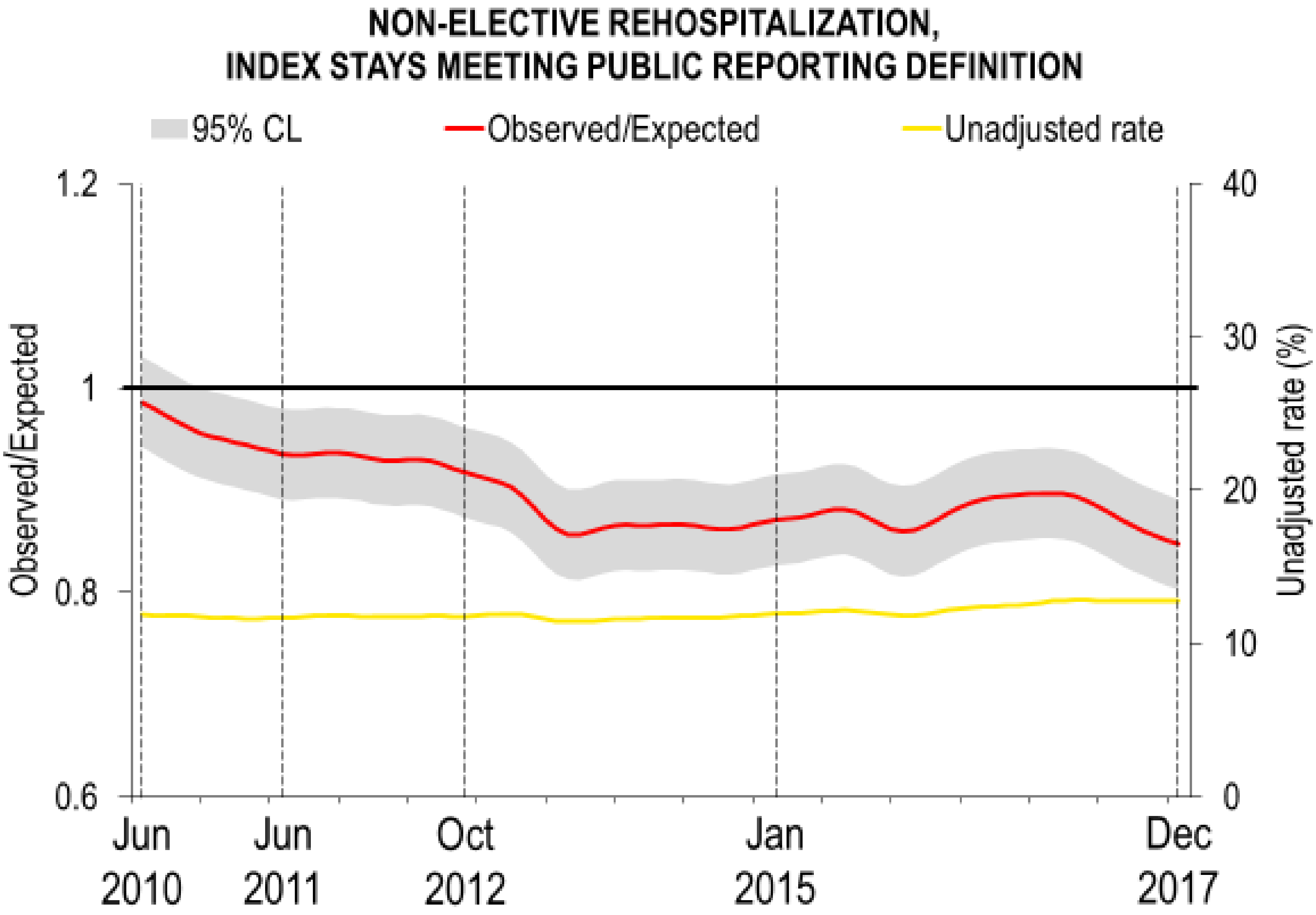

©2019 Escobar GJ et al. *JAMA Network Open*

eAppendix 5. Impact of Denominator Definition on Outcome Capture

Table S5A-1: Patients < 65 years of age				
	DENOMINATOR[a]			
	INPATIENT		***PUBLIC REPORTING***	
Event Type	**6/10 – 5/11**	**1/17 – 12/17**	**6/10 – 5/11**	**1/17 – 12/17**
Hospitalizations	91.3	80.2	66.1	53.8
Multiple hospitalization patients	88.9	77.0	62.3	50.6
COPS2 ≥ 65 hospitalizations[b]	92.4	82.3	71.6	63.6
COPS2 < 65 hospitalizations	91.2	79.7	65.3	51.7
LAPS2 ≥ 110 hospitalizations[b]	97.3	92.3	56.4	47.7
LAPS2 < 110 hospitalizations	91.0	79.3	66.6	54.3
Inpatient deaths	98.5	95.5	9.8	4.2
30-day deaths	97.1	92.7	10.5	5.8
Non-elective rehospitalizations[c]	92.6	82.5	57.0	43.7
Deaths within 30 days of discharge	96.1	88.8	13.5	8.9
Composite outcome[d]	92.9	83.1	53.0	40.8

FOOTNOTES

a Table shows the proportion of outcomes captured when one restricts the denominator to inpatient hospitalizations only (left side) or hospitalizations that met the public reporting definition. The public reporting definition restricts the denominator to patients with continuous health plan membership in the 12 months preceding and the 30 days following hospital discharge, with a maximum gap in coverage of 45 days in the preceding 12 months; it excludes OBS hospitalizations and INP hospitalizations with length of stay < 24 hours from both the numerator and denominator; and it does not include mortality as an outcome. See text and Table 2 for additional details.

b See text, footnotes to Table 1, and Escobar et al. (2013) for a description of the COPS2 and LAPS2 scores. Patients with scores at these levels are very ill patients.

c See text and Escobar et al. (2015) for our definition of non-elective rehospitalization. Hospitalizations for observation are included in the base total.

d The composite outcome is non-elective rehospitalization or death within 30 days of hospital discharge. Hospitalizations for observation are included.

©2019 Escobar GJ et al. *JAMA Network Open*

Table S5A-2: Patients ≥ 65 years of age				
	DENOMINATOR[1]			
	INPATIENT		***PUBLIC REPORTING***	
Event Type	**6/10 – 5/11**	**1/17 – 12/17**	**6/10 – 5/11**	**1/17 – 12/17**
Hospitalizations	89.9	79.2	70.7	59.7
Multiple hospitalization patients	87.3	73.9	64.9	53.7
COPS2 ≥ 65 hospitalizations[2]	91.5	79.6	68.4	60.6
COPS2 < 65 hospitalizations	89.4	79.0	71.5	59.2
LAPS2 ≥ 110 hospitalizations[2]	95.9	88.3	57.8	53.7
LAPS2 < 110 hospitalizations	88.9	77.4	72.9	60.9
Inpatient deaths	97.9	93.1	2.8	1.6
30-day deaths	96.4	89.7	4.5	2.9
Non-elective rehospitalizations[3]	91.8	81.5	58.6	45.9
Deaths within 30 days of discharge	95.2	87.8	6.2	4.4
Composite outcome[4]	92.7	83.1	46.2	36.3

FOOTNOTES

a Table shows the proportion of outcomes captured when one restricts the denominator to inpatient hospitalizations only (left side) or hospitalizations that met the public reporting definition. The public reporting definition restricts the denominator to patients with continuous health plan membership in the 12 months preceding and the 30 days following hospital discharge, with a maximum gap in coverage of 45 days in the preceding 12 months; it excludes OBS hospitalizations and INP hospitalizations with length of stay < 24 hours from both the numerator and denominator; and it does not include mortality as an outcome. See text and Table 2 for additional details.

b See text, footnotes to Table 1, and Escobar et al. (2013) for a description of the COPS2 and LAPS2 scores. Patients with scores at these levels are very ill patients.

c See text and Escobar et al. (2015) for our definition of non-elective rehospitalization. Hospitalizations for observation are included in the base total.

d The composite outcome is non-elective rehospitalization or death within 30 days of hospital discharge. Hospitalizations for observation are included.

©2019 Escobar GJ et al. *JAMA Network Open*
